# Not just two languages: Using variation in language experience to understand how cognitive resources shape syntactic processing

**DOI:** 10.1371/journal.pone.0346505

**Published:** 2026-04-10

**Authors:** Nicholas Sulier, Julio Torres, Judith F. Kroll

**Affiliations:** 1 School of Education, University of California, Irvine, California, United States of America; 2 Department of Latin American & Iberian Cultures, University of California, Irvine, California, United States of America; City University of Hong Kong, HONG KONG

## Abstract

Individuals who learn and use two languages come to that experience in many different ways. Recent studies have shown that to understand bilingualism, it is necessary to characterize the variation in experience that continually shapes the use of the two languages. The current investigation explored the consequences of individual differences in cognitive resources for the processing of syntactic information in two groups of speakers. One group were adults learning Spanish and the other were heritage bilinguals with Spanish as the home language. Both groups were proficient speakers of English. We examined the effects of working memory and cognitive control on syntactic processing, measured by an elicited sentence imitation task. The findings revealed both common and distinct contributions of cognitive resources. Working memory predicted Spanish syntactic processing for second language learners but not for heritage speakers. In contrast, working memory predicted English syntactic processing only for heritage speakers, and this effect was modulated by language dominance. The results for Spanish align with expectations, but the English findings suggest that syntactic processing is shaped not only by proficiency, but also by how the two languages are learned and used together. Cognitive control also showed group-specific effects in response to syntactic complexity: heritage speakers with more reactive control strategies showed better Spanish processing as phrase complexity increased, while L2 learners with more proactive control strategies showed better English processing under increased complexity. Together, these results contribute to our understanding of the effects of language experience on cognitive engagement and shed new light on the dynamics that underlie variation in syntactic processing among bilinguals.

## Introduction

A focus in recent studies of second language (L2) learning and bilingualism is on how variation in language experience influences the engagement of domain general cognition (e.g., cognitive control, working memory) during learning and processing. Beyond addressing misconceptions about the homogeneity of bilingualism, research over the past decade has demonstrated that bilingual cognitive engagement is not uniform but varies with the contexts in which dual language use is embedded [1–4]. These findings highlight the importance of characterizing individual differences when examining how cognition supports language outcomes [5,6].

At the core of this variation is the phenomenon of language co-activation, the idea that a bilingual’s two languages are always simultaneously active in the mind [7], even across differences in script or modality [8–10]. Because both languages are active, bilinguals must flexibly regulate and coordinate them to support successful dual language use. Cognitive control, a cognitive process related to the ability to maintain and update information to guide goal-directed behavior [11], has been shown to play an important role in this regulation, enabling bilinguals’ use of one language with high levels of fluency without inadvertent slips into the wrong language [12,13]. Early empirical evidence for the role of cognitive control during dual language use comes from the finding that control of the first language, the L1, is necessary while using the L2 [14–17], implicating cognitive control as a mechanism responsible for the coordination of two languages. More recent investigations suggest that not only is cognitive control integral to bilingual language use, but that the engagement of this cognitive process reflects individual differences in language experience and also in the demands that the environment places on language users [1].

Frameworks such as the Adaptive Control Hypothesis (ACH) [2] extend this view, proposing that cognitive control mechanisms adapt to the unique interactional contexts in which bilinguals use their two languages. From this perspective, variability in bilingual experience (e.g., how, when, and with whom the languages are used) can drive different patterns of cognitive engagement. However, most of these studies have focused on late bilinguals, and much less is known about how cognitive control operates in other bilingual populations, such as heritage speakers [18]. Furthermore, it remains unclear whether these adaptations also extend to other cognitive mechanisms, such as working memory.

Working memory is a key cognitive process that supports the active maintenance of linguistic representations during both comprehension and production, and research shows that it contributes significantly to language acquisition and processing across a wide array of bilingual groups [19–23]. Importantly, several influential theories of working memory [24,25] suggest that the process of selecting and managing the representations that enter working memory, whether from perceptual experience or long-term memory, is also a vital part of working memory function. For bilinguals, who must manage linguistic representations from two languages, this process is dynamic because the two languages are not always equally available and vary in how they are used. The cognitive demands for bilingual speakers in environments where the two languages are used separately are quite different than those for bilinguals who may switch frequently between the two languages. Yet few studies have considered the role of variation across dual language experience in the engagement of working memory resources.

To address this gap, the present study intentionally examines cognitive control and working memory engagement in two groups of bilinguals with distinct language experiences: 1) adult language learners learning Spanish as an L2, and 2) heritage speakers of Spanish who speak English as a societal language. In the current paper, we take a liberal stance on the term “bilingual” and use it to refer to individuals who have experiences learning and using two languages. We view bilingualism as a spectrum of experience, and this means that individuals with varied levels of proficiency, like adult second language learners and heritage speakers, are bilinguals, albeit on different points of the spectrum. Importantly, although we use the term “bilingual” in a broader way for ease of reference, we also recognize the heterogeneity of bilinguals themselves and aim to reflect this in the detailed categorization of both our groups of interest.

Critically, some recent research has shown that the aggregation of proficient speakers of the same languages can sometimes mask the ways that cognitive resources are recruited to enable language processing. For example, Beatty-Martínez et al. [1] showed that differences in how the two languages were used for similarly proficient speakers living in distinct interactional contexts appeared to determine the cognitive resources that were recruited to support language processing. This work and others [18,26,27] provides an empirical basis for the concern that grouping bilinguals without characterizing their unique experiences can obscure meaningful variation. In the current study, we directly address this by exploiting variation in bilingual experience to ask how working memory and cognitive control support syntactic processing in both languages. While most prior studies have focused on lexical production and comprehension, the present work shifts attention to syntactic processing to test whether these same cognitive resources are engaged differently when demands extend beyond the word level. We hypothesize that both groups of bilinguals will recruit working memory and cognitive control during syntactic processing, but that their distinct language experiences may modulate how these cognitive resources are engaged.

In what follows, we first review background on adult language learners and heritage speakers to understand the nature of their language experience. We then clarify how cognitive control and working memory may function as related but distinct cognitive resources. Prior research on cognitive control and bilingual language processing is then reviewed, with a focus on highlighting why these differences are important to consider when we examine how cognition is engaged to support dual language use. Finally, detailed information will be shared about the relationship between working memory and language use more generally, followed by evidence supporting the relationship between working memory and syntactic processing, setting the stage for the current study.

### Adult Language Learners and Heritage Speakers

Adult language learners and heritage speakers represent two groups whose bilingual experiences differ in important ways. Adult language learners (also referred to as late sequential bilinguals) are individuals who learn an additional language (L2, L3, L4, etc) in addition to their L1, and this process typically begins well beyond early childhood. For example, in the United States, an adult learner might be a native or L1 speaker of English learning Spanish as an L2 at a secondary or post-secondary institution. Although adult learners are typically assumed to be immersed in the L1 environment, they may also be learning the L2 under conditions of L2 immersion, a context that characterizes adult learners studying abroad or learning following immigration to the L2 context. In contrast, heritage speakers in the U.S. are individuals who acquire a non-English minority language at home while also being exposed to English as the societal language, which can occur not only in educational contexts, but also with friends, relatives, media, and work. They are considered bilingual in English and their home language, yet their command of both languages can vary based on any number of factors, including the opportunities they have to use and maintain the home language as well as schooling [28,29]. Importantly, heritage speakers can also be considered native speakers in both of their languages. For the purpose of clarity, any reference in the current paper to the home/heritage language acknowledges this and does not inadvertently suggest that they are not native speakers of their heritage language. L2 learners and heritage speakers differ in their experience at many levels (i.e., when, and why a language is acquired, how often it is used, where it is used, and with whom). They may also differ in language dominance. We consider language dominance as a multidimensional construct reflecting a bilingual’s relative ease of access to one language over the other. Language dominance can be shaped by factors such as proficiency, age of acquisition, frequency of language use, and language attitudes. It can be measured, for example, by tools such as the Bilingual Language Profile questionnaire [30,31]. Bilinguals’ language dominance can shift over a bilingual’s lifespan, primarily due to changes in the linguistic environment caused by life events such as immigration and schooling [32]. But language dominance can also shift dynamically, especially for heritage speakers, as individuals move across different interactional contexts within the course of even a single day [18]. We predict that L2 learners in classroom contexts will predominantly be L1-English dominant, whereas heritage speakers will demonstrate greater variability in language dominance, despite typically being dominant in English as the societal language.

Variation in language experience, particularly in language dominance, gives us the opportunity to address the important question of how domain-general cognitive processes, like working memory and cognitive control, interact with language knowledge and experience. Addressing this question will help add important information to ongoing theoretical discussions on the role of domain-general cognition during language use. For example, modular conceptualizations of the language system posit a separation between core linguistic and domain-general processes [33], which can lead to a more dichotomous understanding of the relationship between language and cognition. Under a modular view, domain-general cognition is recruited only when core linguistic processes fail to meet task demands. Yet, more recent research shows that language processing engages cognitive processes in interactive ways [26,34], suggesting that the relationship between language and cognition may be more dynamic and interconnected. By examining cognitive engagement in two distinct bilingual groups, this study seeks to clarify the interconnected relationship between language and cognition. Evidence for this can further inform discussions of established theories, particularly of working memory, and how they might be expanded to account for this dynamic relationship.

### Cognitive control and working memory

In the current study, we make a distinction between cognitive control and working memory. Cognitive control is the ability to maintain and update internal representations of information needed to guide context-relevant, goal-directed behavior [11]. Because our focus is on adaptive cognitive engagement during bilingual language use, we also frame cognitive control in terms of its strategic, temporally dynamic implementation, as outlined in the dual mechanisms of control (DMC) framework [35]. The DMC framework posits that there are two distinct operating modes: proactive control and reactive control. Proactive control can be conceptualized as a form of ‘early selection’ in which goal-relevant information is actively maintained in a sustained manner, before the occurrence of cognitively demanding events, to optimally bias attention, perception and action systems in a goal-driven manner. By contrast, in reactive control, attention is recruited as a ‘late correction’ mechanism that is mobilized only as needed, in a just-in-time manner, such as after a high interference event is detected.

In contrast, we define working memory as “those mechanisms or processes that are involved in the control, regulation and active maintenance of task-relevant information in the service of complex cognition” [36, p. 450]. Because the focus of the current study is on syntactic processing, which requires simultaneous processing and storage, we assess working memory using an operation-span task [37], which measures an individual’s ability to store information while engaged in additional processing tasks. This “complex” span task engages the executive control component of working memory—the component responsible for coordinating storage and processing demands and managing interference. Although this executive component shares important features with cognitive control (e.g., attention regulation, inhibition of distraction), it is typically situated within a capacity-based framework of working memory rather than the more strategically oriented DMC framework that is central to the current study. This distinction is particularly important in bilingual contexts, where working memory capacity has been shown to support the management of competing linguistic representations [21] and real-time processing of a L2 [22]. Given this conceptual overlap yet functional distinction, the next section will further clarify how working memory and cognitive control relate to, and differ from, one another.

While working memory and cognitive control have each been investigated during language processing, they rely on different underlying mechanisms. Working memory refers to the ability to actively maintain and manipulate information, and its contribution to language is largely capacity dependent. Working memory supports processes involved in both L1 and L2 acquisition [22,38], and studies on adult language learning consistently report that individuals with higher working memory capacity achieve better learning outcomes [21,39,40]. For heritage speakers, working memory has also been linked to writing processes, where modality shapes engagement [23]. The underlying assumption is that working memory is recruited whenever linguistic input must be stored, integrated, or manipulated; thus, greater capacity translates directly into more successful processing and learning.

However, working memory effects are not uniform across all bilinguals. For more proficient or skilled bilinguals, strong working memory effects often diminish, as high levels of language experience and automatization reduce the need to actively maintain and manipulate linguistic information [22]. Heritage speakers, while generally skilled bilinguals, vary widely in their use of each language across different contexts. This variability matters: more frequent or mixed use may place heavier demands on working memory, whereas more compartmentalized use may reduce working memory’s importance.

In contrast to working memory, cognitive control is not primarily about capacity but about strategy—how individuals regulate attention and behavior to meet language processing demands. Cognitive control involves selecting, inhibiting, and switching between competing representations. In bilinguals, this often takes the form of strategic control, as posited by the DMC framework. Although working memory and cognitive control are distinct, they are interdependent. Studies show that working memory capacity constrains the effectiveness of control strategies: high working memory capacity predicts more efficient proactive control, likely because predictive planning requires sufficient resources [41–43, but see 44].

As mentioned previously, we examine cognitive control and working memory using different tasks. Cognitive control is assessed with the AX Continuous Performance Task (AX-CPT), which is designed to capture the strategic deployment of proactive and reactive control in line with the dual mechanisms of control framework [35]. Working memory is measured with the operation span task (OSPAN), a complex span task that requires participants to maintain items in memory while processing unrelated information. Although complex span tasks necessarily engage executive processes (e.g., resisting distraction, switching between storing and processing), they are widely interpreted as reflecting executive working memory capacity, or the amount of information that can be actively maintained and manipulated during concurrent processing [45,46]. In this way, the OSPAN primarily captures the capacity-based mechanism of working memory, while the AX-CPT emphasizes the strategy-based deployment of control, allowing us to disentangle the complementary but distinct contributions of these two cognitive processes to bilingual syntactic processing.

### Cognitive control during bilingual language processing

Research indicates that different bilingual experiences and interactional contexts play a critical role in shaping how bilinguals recruit cognitive control strategies during language processing. Beatty-Martínez et al. [1] compared three groups of Spanish-English bilinguals, all highly proficient in both languages across different contexts that varied in the linguistic and cultural expectations for using both languages. One group in Granada, Spain, used Spanish and English separately. Another in San Juan, Puerto Rico, used both languages almost interchangeably in their daily lives. A third group were immersed in State College, Pennsylvania, an English dominant environment context where few other people spoke Spanish. To measure cognitive control, Beatty-Martínez et al. used the AX-CPT [47] to determine whether lexical production was differentially modulated by cognitive control as a function of the context in which bilinguals lived. The results showed that lexical production, indexed by a picture naming task, differed across the three groups. Most notably, the bilinguals in Pennsylvania, who were immersed in English (their L2) appeared to rely heavily on proactive control strategies to maintain proficiency in Spanish (their L1). The bilinguals immersed in an L1 environment in Spain, with separate use of the two languages, were more reliant on reactive control processes. Those in the more cooperative environment in Puerto Rico demonstrated no clear reliance on either control strategy. These results are important because they demonstrate that cognitive control plays an important role during lexical selection and use.

In a recent study, Hernandez Santacruz et al. [18] examined how Spanish-English heritage speakers in Southern California, a bilingual group who often use both their home and societal languages differently within the course of a day, engaged cognitive control mechanisms. Like Beatty-Martínez et al. [1], the study used picture-naming tasks in Spanish and English and also the AX-CPT but varied the order in which bilinguals named pictures in each language. Previous research has shown that the order in which the two languages are spoken may reflect the way they are regulated and how they engage cognitive resources [48,49]. Hernandez Santacruz et al. found that heritage speakers in the Spanish-naming first condition relied more on proactive control strategies during picture naming, consistent with Beatty-Martínez et al.’s bilingual group in the U.S. However, this pattern did not hold for participants in the English-naming first condition when switching from English, the societal language, to Spanish, the home language. Specifically, heritage speakers in the English-naming first condition exhibited no effects of cognitive control on picture naming accuracy. These findings suggest that heritage speakers’ frequent opportunities to code switch, as a natural part of their bilingual interactional context, may shape how they recruit cognitive resources.

Cognitive control is also relevant for L2 learners in a classroom context. Luque and Morgan-Short [50] examined intermediate Spanish learners using two executive control tasks –– Flanker and AX-CPT –– in addition to measures of L2 proficiency. Proficiency measures included a written test targeting vocabulary and grammar, a timed semantic verbal fluency task requiring word production, and an elicited imitation task in which participants repeated sentences aloud. The findings for performance on the AX-CPT showed that reactive cognitive control patterns were significantly associated with higher L2 proficiency composite scores. Although the elicited imitation task was not explicitly examined in the regression analysis carried out in the study, there was a significant negative correlation between EIT performance and speed of processing in the AX-CPT task, suggesting that faster speed of processing was related to better EIT performance.

Taken together, these studies indicate that different bilinguals, across various interactional contexts, rely on cognitive control strategies to regulate and coordinate the management of both languages during language processing tasks, particularly during activation of lexical information. However, the recruitment of these cognitive control strategies is not uniform, as factors such as bilingual experience and interactional context appear to influence which cognitive control strategies are engaged. At the same time, it is important to note that much of this research has focused heavily on lexical production measures such as picture naming and verbal fluency. While informative, this emphasis leaves unexplored whether cognitive control engagement during other forms of language use, like syntactic processing, is similarly impacted by variation in language experience.

By examining syntactic processing in the current study, the boundary conditions of these effects can be further clarified. In addition, although the studies by Beatty-Martínez et al. [1] and Hernandez Santacruz et al. [18] examined the relationship between cognitive control and language processing in both the L1 and L2, the study by Luque & Morgan-Short [50] only focused on the L2, which leaves a gap in our understanding of how cognitive control engagement might varying between the L1 and L2 in adult language learners. By examining both languages in adult L2 learners and heritage speakers in the current study, we have the opportunity not only to address this gap but also ask whether cognitive control engagement shifts even for a language in which both groups demonstrate similar levels of proficiency. Finally, while past studies make clear that cognitive control engagement can vary across distinct bilingual groups, is it less clear whether adaptive engagement extends to working memory. In the following section, we review past research on working memory and language processing more generally before providing more detail on syntactic processing, the focus of the study.

### Working memory during language processing

Meta-analyses have demonstrated that both L2 comprehension and production are supported by working memory. For example, Linck et al. [21] examined data from 79 studies involving over 3000 participants, with results indicating that working memory is positively associated with both L2 processing and proficiency outcomes, with an estimated population effect size (ρ) of .255. Similar correlations were found for comprehension and production outcomes, suggesting that working memory is relevant to both processes. Finally, results indicated that the executive control component of working memory (often operationalized by complex span tasks) was more strongly related to L2 outcomes than the storage component (phonological working memory), which showed attenuated but still significantly positive effect sizes.

A positive relationship between working memory and language processing has been reported for Spanish-English bilinguals and for monolingual English speakers, but taking different forms for the two groups. Navarro-Torres et al. [34] asked whether working memory mediated variation in a-adjective usage (*asleep, afraid*), which, unlike typical adjectives (*sleepy, frightened*), tend to resist attributive use. They found that for both monolinguals and bilinguals, the tendency to use a-adjectives attributively or non-attributively was modulated by individual differences in working memory. For bilinguals, however, a-adjective use was additionally modulated by an interaction between working memory and category verbal fluency in the dominant language (English), revealing an interactive role of domain-general and language-related mechanisms that enable regulation of competing alternatives. Ultimately, these results illustrate that while working memory appears to support language use at a general level across all language users, bilinguals reveal variation in how it is engaged, which motivates the current study’s focus on working memory engagement during syntactic processing.

Finally, working memory has also been found to support bilingual language use in heritage speakers, a population of focus in the current study. Torres [23] investigated the relationship between pausing and revision behaviors in Spanish-English heritage speakers. Importantly, these writing processes were examined in both the heritage language (Spanish) and the societal language (English). Individual differences in working memory were captured using an operation-span task (OSPAN). The primary result was that heritage language writers with higher working memory had significantly longer pause times within words, independent of the language they were using (Spanish or English). However, think aloud data revealed that the situation was more complex. Specifically, the think aloud data revealed that, in the Spanish task, pausing behaviors were more focused on linguistic encoding issues, particularly the retrieval of lexical items from long-term memory. In the English task, pausing was related to the planning and organization of content. Critically, these results suggest that, while working memory is engaged by heritage speakers to support language use in both the home language and the societal language, the factors underlying its engagement may vary to suit the needs and demands of particular speakers.

Taken together, the previous studies illustrate that working memory is a vital cognitive process that supports many forms of comprehension and production in both adult L2 learners as well as bilinguals from varied backgrounds. The results from both Navarro-Torres et al. [34] and Torres [23] show that working memory engagement varies across different groups (bilinguals vs monolinguals) as well as languages (heritage language vs societal language). While these studies illustrate important results related to the relationship between working memory and language use, there are several limitations that the current study aims to address. First, while it seems clear that working memory engagement can vary between monolinguals and bilinguals [34], these results may have been driven by the differences between each group’s language experiences, and the differences in cognitive demands associated with those experiences. It is less clear how working memory engagement might vary between two bilingual groups who speak the same languages yet do so with varied patterns of use at different developmental stages (i.e., adult learners and heritage speakers). Additionally, while the studies by Navarro-Torres et al. [34] and Torres [23] examined both L1 and L2 language use in their bilingual groups, studies examining working memory in adult learners are typically constrained to working memory effects on the L2, not the L1. This leaves an unanswered question as to whether working memory engagement in a L1 can change in adult L2 learners due to the demands of becoming bilingual. Finally, unexplored in these studies is syntactic processing, the focus of the current study. Below, we will briefly elaborate on the relationship between syntactic processing and working memory and present and justify the use of an Elicited Imitation Task (EIT) as a measure to operationalize this linguistic ability.

### The relationship between working memory and syntactic processing

Understanding L1 or L2 processing in real time relies on the ability to keep track of who did what to whom during sentence and discourse comprehension [51]. To achieve this, individuals must actively process and determine how words and morphemes combine to form larger units, such as sentences, a process we refer to as syntactic processing. Syntactic processing during both listening and reading relies heavily on working memory, and evidence for this comes from experimental studies on ambiguity resolution and gender agreement [52–54], applied linguistics studies on speaking and reading [55] and studies examining brain activity using event-related potentials [56,57]. While the importance of working memory to language processing is not contested, there is debate surrounding the different theoretical conceptualizations of this cognitive process. While a full review of this debate is beyond the scope of this paper, we will touch briefly on two of the dominant approaches, as their distinct conceptualizations of working memory are relevant to the current study on bilingual language processing.

One dominant approach to working memory are capacity-based views [58], which are broadly compatible with the well-known multiple-component models [59]. These accounts theorize that there is a dedicated working memory component that has a capacity that differs from one individual to another, which results in individual differences in L1 and L2 language abilities. When extended to syntactic processing in L2 learners or bilinguals, these models would predict that differences in syntactic processing may be driven by one’s ability to maintain words/phrases in working memory; less capacity would result in a reduced ability to process syntactic information.

In contrast, other models characterize working memory as the ability to allocate attention to bring task-relevant information in and out of the focus of attention [24,25]. Under these models, working memory is a form of attention which selects and holds information to support complex cognitive operations. Critically, however, these models also suggest that managing interference is a vital part of working memory; only information relevant to one’s current goals must be admitted into working memory as to avoid interference. Thus, under these accounts, individual differences in working memory are driven by both the ability to control attention to select and hold representations in working memory, but also by the ability to manage interference from competing representations. When extended to syntactic processing in L2 learners and bilinguals, these models predict that differences in syntactic processing ability should be accounted for by the ability to select and hold relevant linguistic information in working memory, and also by the ability to manage interference from competing linguistic information.

In short, capacity-based views propose that successful syntactic processing depends primarily on how much information can be held in working memory at once, whereas controlled attention accounts emphasize an individual’s ability to direct attention toward relevant information and manage interference from competing inputs. Importantly, these distinctions become especially relevant for bilinguals, who must both maintain and update linguistic information in working memory and also regulate interference between their languages. This added layer of attentional control is likely to vary across bilingual groups whose distinct language experiences create different inhibitory demands. For example, L2 learners and heritage speakers are typically highly proficient in English, yet they differ significantly in their proficiency in Spanish. When processing English, both groups must use working memory to support the processing of syntactic information. However, because heritage speakers also exhibit advanced proficiency in Spanish, they experience greater co-activation of the non-target language, creating increased competition that requires additional controlled attention to regulate. In contrast, L2 learners’ lower Spanish proficiency leads to less co-activation and therefore fewer interference-related demands, though they may still be limited by the overall capacity available to maintain linguistic information during processing.

The current study is primarily motivated by and framed within the controlled attention account of working memory. Our research design, which compares distinct bilingual groups (heritage speakers and L2 learners), is specifically intended to investigate how differences in language experience influence the demands of working memory (framed as attentional control and interference management) during syntactic processing. While performance on the EIT may be influenced by general working memory capacity (as per the capacity-based view), the central hypothesis we test—concerning the role of interference management in bilinguals—is a specific prediction of the controlled attention model. It is important to note, however, that the operation span task we use in the current study does not provide a pure measure of attentional control versus capacity. As a complex span task, it taps into both processing and storage components and thus does not allow for a clear and explicit distinction between the two theoretical frameworks. Our goal is to use the task as a general index of individual differences in working memory, with the bilingual group comparisons offering the primary insight into attentional control and interference management.

These theoretical distinctions motivate the present study, which compares cognitive engagement in L2 learners and heritage speakers during syntactic processing, measured by an elicited imitation task or EIT [60]. The EIT is ideal for this comparison because it potentially places demands on working memory. In the EIT, participants repeat sentences that gradually increase in length (7–17 syllables) and syntactic complexity, including structures that are challenging for learners (e.g., Spanish reflexive *se*, subjunctive; see Methods). In this way, participants must hold a sentence in mind and then reproduce it, implicating working memory engagement. Importantly, unlike tasks that require advanced speech planning or high literacy, the EIT is accessible to both L2 learners and heritage speakers, making it a fair tool for cross-group comparison. The task is also sensitive to syntactic knowledge, since accurate reproduction requires parsing and reconstructing sentence structure, providing a measure of syntactic processing in each language. By using the EIT, we can examine how working memory (and cognitive control) support syntactic processing across bilingual groups with differing language experience in Spanish, providing a controlled and theoretically informative comparison.

### Summary

Cognitive control and working memory are distinct yet complementary processes that support bilingual language use. While research shows that bilinguals rely on cognitive control to manage both languages, the strategies employed vary with experience and context, and it remains unclear whether these effects extend beyond lexical production to syntactic processing. Similarly, working memory is critical for comprehension and production across bilingual and monolingual speakers, but how its engagement differs during syntactic processing in bilinguals who share the same languages yet have distinct experiences—such as adult L2 learners and heritage speakers—remains an open question. Below, we share our predictions before introducing the current study, results, and discussion.

### Predictions

We predict that working memory and cognitive control will support syntactic processing differently for heritage speakers of Spanish and for L2 learners of Spanish, reflecting their distinct language experiences. First, we predict that the adult learners will reveal the effects of working memory on the processing of the L2 they are learning (Spanish). Those learners with better working memory will be better able to process the target language due to their increased ability to encode, maintain, and retrieve linguistic information in the L2. While there may be individual differences in the processing of the L1 (English) [61–63] we expect them to be reduced. Next, we predict that heritage speakers will reveal the effects of working memory when processing their less-dominant language. Specifically, we predict that English-dominant heritage speakers with higher working memory will be able to better process the heritage language (Spanish), and that Spanish-dominant heritage speakers with higher working memory will be better able to process the societal language (English). These working memory effects in heritage speakers may also be driven by the amount of interference they must manage, aligning with controlled-attention models of working memory.

For cognitive control, we predict that learners, who are primarily exposed to Spanish in the classroom, are likely to maintain language separation, promoting a reactive control pattern during syntactic processing in Spanish, similar to the finding reported by Luque and Morgan-Short [50]. The pattern of cognitive control engagement in English for L2 learners may depend on the degree to which their English is regulated to enable the use of the less dominant Spanish. For heritage speakers, cognitive control predictions are less straightforward because their language use is more varied and context dependent. According to the Adaptive Control Hypothesis, frequent language switching and engagement in multiple interactional contexts can promote proactive control, whereas more separated use may encourage reactive control. Thus, we might expect heritage speakers to show a flexible pattern: proactive control could emerge during Spanish processing when cross-language interference is high, while reactive control could dominate in English if language use is more stable and separated. This aligns with past work showing that bilinguals adapt their control strategies to the demands of their interactional contexts [4]. Finally, we expect that the effects of working memory and cognitive control may be more likely to be revealed when the linguistic demands of the language processing task are increased [64].

### The Current Study

The current study examined the effect of working memory and cognitive control on syntactic processing ability in English and Spanish, as measured by an oral elicited imitation task [60], in adult learners of Spanish and Spanish-English heritage speakers. Critically, we investigated individual differences in working memory, measured by the complex operation span task [65] and cognitive control engagement, measured by the AX-Continuous Performance Task [16,66]. Both groups of bilinguals were highly proficient in English, the societal language, but differed in their use of Spanish. We considered how each of these cognitive factors might modulate syntactic processing in both English and Spanish. An important goal is to move beyond binary classifications of bilinguals as simply being early or late acquirers with resulting proficiency that is high or low (see [67] for a recent discussion of age effects in second language learning).

## Methods

### Ethics statement

Each participant who gave consent to participate in the study was tested online. Participants gave informed written consent as the researcher observed on zoom. Consent was documented online using an uploaded .pdf version of the consent form. All procedures were performed in compliance with the principles expressed in the Declaration of Helsinki and were approved by the IRB (IRB # 2019–5424). The current study was reviewed and approved by the UC Irvine: Office of Research Institutional Review Board before the study began. The study did not include minors. The recruitment period began 11/12/2021 and ended 06/4/2025.

### Participants

The current study tested 48 learners of Spanish and 36 heritage speakers of Spanish recruited from a mid-size public university in the western United States. Learners were recruited from intermediate Spanish courses while heritage speakers were recruited from the university’s human subject lab pool. Based on reported information from the Bilingual Language Profile Questionnaire (BLP), 34 learners identified as female and the mean age among these participants was 21 (*SD* = 3.9). For heritage speakers, 32 identified as female and the mean age among these participants was 20.1 (*SD* = 1.9).

### Language background & proficiency measures

#### Bilingual Language Profile Questionnaire (BLP).

Prior to the experimental session, participants completed the Bilingual Language Profile Questionnaire (BLP) [31] online through Google Docs. The BLP was used to assess language history, proficiency, use, and attitudes in English and Spanish through self-reports. These combined measures were used to calculate participants’ relative language dominance score. Language dominance was calculated in the following way: First, a score for English and Spanish was calculated for each of the four modules (language history, use, proficiency and attitudes). These subcomponents are briefly described below:

**Language History** Measures early life exposure to a language, including age of acquisition, length of residence in different language environments, and language use during childhood. **Language Use:** Captures the frequency and domains in which a language is used currently (e.g., at home, at work, with friends, for media). **Language Proficiency:** Assesses a self-reported rating of skills in speaking, listening, reading, and writing for each language. **Language Attitudes:** Reflects subjective feelings and perceptions about each language, including emotional attachment and cultural identification.

After applying adjustment factors, module scores were then summed to produce a total global score for English and Spanish. Finally, the language dominance index was calculated by subtracting the global Spanish score from the English score, resulting in a continuous index ranging from −218 to +218. A score near zero indicated no clear dominance in either language, while a more positive score reflected more English dominance, and a more negative score reflected more Spanish dominance. Critically, the BLP captures many dimensions of dominance, including proficiency, and while scholars agree that language dominance and proficiency overlap, they are considered distinct constructs [68]. In addition to the questions already included on the BLP, participants were also asked to share information on their experience with languages other than English or Spanish. The primary purpose of these additional questions was to characterize the learner population.

It is important to note that this assessment relies on self-report data, which, as acknowledged by past research [69], can be subject to individual biases and potential misalignment with objective proficiency measures. However, the BLP provides a comprehensive and efficient assessment of various aspects of a speaker’s linguistic profile, including usage, history, and attitudes. Given our focus on comparing distinct bilingual groups with differing language experiences, the BLP’s broad scope was deemed appropriate for this study. We acknowledge that future research would benefit from incorporating a multi-method approach, combining self-report with objective measures to provide a more comprehensive picture of language dominance.

#### Spoken Elicited Imitation Tasks (EIT).

Participants completed the EIT in both English and Spanish. Both tests were developed by Ortega et al. [70] and were administered online using the Gorilla Experiment Builder (www.gorilla.sc). The EIT was originally developed as a measure of oral proficiency, as it taps into several sub-components of proficiency at once, including processing, storage, comprehension, and production. Critical to the current study, it also involves the encoding of sentences of increasing length and syntactic complexity, a linguistic skill which has been related to the memory system [71] and domain-general attentional resources [72]. For these reasons, the current study will consider the EIT as a measure of syntactic processing, while also recognizing its more popular utility as a measure of oral proficiency.

The EIT requires participants to listen to and repeat a series of 30 sentence stimuli. Sentences can range from 7 to 19 syllables in length for English and 7–18 syllables for Spanish. Sentences also contain syntactic structures that are typically difficult for most individuals, particularly learners (e.g., in Spanish, *reflexive se, subjunctive*). The position of such structures varied within stimuli of different lengths. A more detailed distribution of syllable length is included in Table 45 and Table 46 in S1 Appendix. An example of this is shown below with subjunctive in Spanish (both sentences contain subjunctive but vary in syllable length).

1. Syllable Length (9)

**Table:** 

Dudo	que	sepa	manejar	muy	bien
Doubt-1sg	that	know-1sg-subj	drive	very	well

‘I doubt they drive very well.’

2. Syllable Length (16)

**Table:** 

Le	pedí	a	un	amigo	que	me	ayudara	con	la tarea
IO	ask-1sg-prt	to	a	friend	that	1sg-DO	help-3SG-subj	with	the homework

‘I asked a friend to help me with the homework.’

In order to confirm that the EIT stimuli were indeed increasing in syntactic complexity, correlations were computed between the English EIT item number and several measures of syntactic complexity (shown in Table 47 in S1 Appendix). In the EIT, an increase in item number reflects an increase in syllable length, meaning the higher the item number is, the longer the sentence is in terms of syllable length. These syntactic complexity metrics were computed using the NeoSCA tool [73] which are based off length-based “global” measures described by Norris & Ortega [74]. The analysis of the English EIT items revealed significant positive correlations between item number and several syntactic complexity measures, including but not limited to clauses per sentence and dependent clauses per clause. This supports the interpretation that the stimuli increase in both syllable length as well as syntactic complexity.

Participants were given written instructions in English to try and repeat exactly what they had heard. After being given practice sentences, the following assessments in both English and Spanish consisted of 30 sentences. After each sentence, there was a 1-s pause, followed by a 0.5-s tone that cued the oral repetition of the sentence. Participant responses were recorded and saved online using Gorilla. Scoring criteria followed Ortega [60] and were based on the quality and quantity of the sentence that was repeated (See Table 44 in S1 Appendix for scoring criteria). The maximum score on the test was 120.

### Individual differences measures

#### Working Memory (Operation-Span Task).

This task was an automated version of the Operation-Span Task [37] and was carried out on the Gorilla Experiment Builder (www.gorilla.sc). In this task, participants were asked to solve simple math operations while maintaining sets of letters in memory for later recall. For example, participants first saw the math problem (e.g., (9 + 5) * 1 = 15?), reported whether the solutions were correct or incorrect using a mouse click, and then were shown a letter on the screen (“w”). Participants were shown 15 letter sequences that spanned from three to seven letters. After a full sequence was presented, participants were asked to recall the letters presented in order by typing them one by one using their keyboard. The OSPAN score was calculated as the total number of perfectly recalled sets. We did not filter scores based on accuracy of the math operation problems as it has been found not to impact the validity of working memory tasks [75].

#### Cognitive Control – AX Continuous Performance Task (AXCPT).

In this study, we used the AX Continuous Performance Task as a measure of cognitive control [16,66]. The AX-CPT is a nonlinguistic task developed to study variability in the use of proactive (e.g., goal maintenance, conflict monitoring, and interference suppression) and reactive control (e.g., response inhibition). The version of the task used here included distractors due to evidence suggesting that AX-CPT tasks with distractors are more sensitive to individual differences in young healthy adults as compared to other versions of the task. The task was carried out on the Gorilla Experiment Builder (www.gorilla.sc). Participants were presented with a sequence of letters for 300 ms each in the center of a white screen. The letters were displayed on a cue-probe basis so that there was 4900 ms between presentation of the cue and probe. The intertrial interval was 1000 ms. Participants were asked to maintain the cue in memory (which could either be the letter “A” or “B”) until they saw the probe (which could be “X” or some other letter, but not “A”, “K” or “Y”). Whenever they saw the “A” cue followed by the “X” probe, they were asked to respond “yes” by pressing “m” on their keyboard. In any other possible cue-probe combination, they were asked to respond “no” but pressing “z” on their keyboard. In the interval between the cue and probe, participants saw three distractor letters (“F”, “M”, and “D”) presented in black. Distractors were presented for 300 ms with a 1000 ms interval between letters. Participants were asked to respond “no” to each distractor. The task began with 10 practice trials including all 4 possible experimental conditions: AX (a “A” cue followed by a “X” probe) BX (a “B” cue followed by a “X” probe) AY (a “A” cue followed by a non-X probe) and BY (a “B” cue followed by a non-X probe). Participants were provided feedback on the practice trials. The experimental block was comprised of 100 trials in which AX trials occurred 70% of the time and each of the other conditions occurred 10% of the time. Following Braver et al. [47], accuracy (percent correct) and mean reaction time (RT) data were computed for each of the four trial types (AX, AY, BX, BY). An additional composite measure was used using the two trial types (AY, BX) that are the most salient measures of the different aspects of context processing ability. This behavioral shift index (BSI) was computed as (AY – BX)/(AY + BX). The index was calculated for errors, RTs, and the sum of errors and RTs. A correction was made for trials where errors were equal to zero (error + 0.5)/(frequency of trials +1). Higher BSI scores indicate greater proactive control. In the current study, we interpret a higher BSI score (more proactive control) to represent greater reliance on context-driven processing. In contrast, a lower BSI score will be interpreted as less reliance on context-driven processing, and therefore greater reliance on reactive control processes.

### Procedure

Each participant who gave consent to participate in the study was tested online. Participants gave informed written consent as the researcher observed on zoom. Consent was documented online using an uploaded.pdf version of the consent form. All procedures were performed in compliance with relevant laws and institutional guidelines and were approved by the IRB (IRB # 2019–5424). Prior to beginning the tasks, each participant met with a researcher on Zoom to receive instructions and task information. The researcher remained on zoom in case the participant had additional questions about task instructions. Participation in the study took place in one experimental session that lasted about 1 hour. The participants were given an opportunity to take a break halfway through the session. Prior to arriving at the virtual experimental session, participants completed the language background questionnaire (BLP). After being given instructions, participants completed the AX-CPT, EIT (English), O-SPAN task, and the EIT (Spanish). Participants were compensated with either course credit or $15/hour.

### Data analysis

Statistical analyses were performed with linear mixed-effects models using the lme4 software package [76] in the R programming environment [77]. For each quantitative variable, outliers were treated using a robust statistical approach to maintain sample size. Specifically, winsorization was employed to cap extreme outlier values with the nearest non-outlier value. This method allowed us to reduce the influence of extreme values without discarding any data. Trial-level performance on the English and Spanish EIT was examined following a maximal procedure as recommended by [78,79]. To address concerns regarding group-specific effects and interactions, separate models were constructed for each group (L2 learners and heritage speakers). Fixed effects included working memory, cognitive control, and dominance. Interactions were included between working memory and dominance and cognitive control and dominance. Random effects included by-participant and by-item random intercepts. Examples of each maximal model for English and Spanish are shown below:

$\begin{array}{cc} & \text{English EIT Model}:lmer(EITEnglishScore\;\sim \;OSPANz*Dominancez\;+\;BSIz*Dominancez+(\text{1}|Participant)\;\;\;\\ & +(\text{1}|EITEnglishToken),data\;=\;LMER\_Learner,REML\;=\;FALSE) \end{array}$

$\begin{array}{cc} & \text{Spanish EIT Model}:\;lmer(EITSpanishScore\;\sim \;OSPANz*Dominancez\;+\;BSIz*Dominancez\\ & \;\;+(\text{1}|Participant)+(\text{1}|EITSpanishToken),data\;=\;LMER\_Learner,REML\;=\;FALSE) \end{array}$

To address the potential effects of different aspects of language experience, follow-up analyses were conducted. Specifically, new models were built for each group, replacing the composite dominance score with one of the four subcomponents from the BLP: Language History, Language Use, Language Proficiency, and Language Attitudes. Since these subcomponents are derived for both English and Spanish, this involved constructing models for each component and each language within each group. The goal of this exploration was to directly test which specific dimensions of language experience might modulate the effects of the cognitive variables (working memory and cognitive control) on syntactic processing. The structure of these models remained the same as the primary models, with the only change being the replacement of the composite dominance score with one of the BLP subcomponents. Finally, it is important to note that interpretations based on these models will be made with caution given that the subcomponent scores are derived from a relatively small set of questions in the BLP.

## Results

We first present results that provide an overview of the characteristics of the bilinguals in the current study and then consider the relationship between the individual difference measures and syntactic processing. Results from linear mixed-effects models on syntactic processing will then be described; these models examined the contributions of working memory and cognitive control on syntactic processing. We finally consider how the linguistic demands in language processing may be important in understanding how cognitive resources are engaged for the two languages.

### English and Spanish syntactic processing

**Fig 1:**
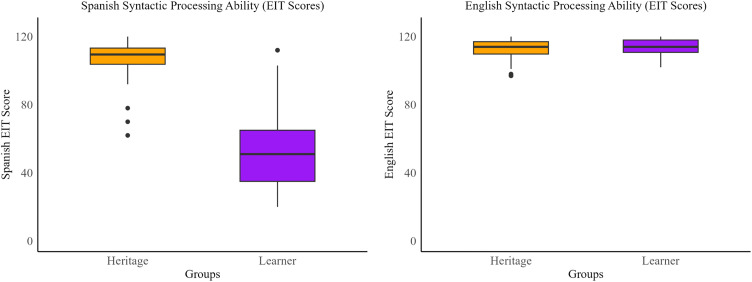
Group level Spanish and English EIT performance.

We first examined differences in the two groups in their ability to perform the EIT task in each language, a measure that has been taken to reflect syntactic processing. Results revealed that learners and heritage speakers performed similarly on the English EIT. However, there were significant differences in Spanish syntactic processing between learners and heritage speakers. These results are shown in Table 1 and Fig 1.

**Table 1:** Descriptive Table.

	**Participant Group**		**p-value** ^*2*^
	**Heritage Speakers**, n = 36^*1*^	**Learners**, n = 48^*1*^	
English AoA (yrs)	3.61 (3.11)	0.79 (2.36)	***p* < 0.001**
Spanish AoA (yrs)	2.00 (4.79)	12.63 (4.12)	***p* < 0.001**
OSPAN (Max 75)	39.83 (16.74)	40.85 (11.90)	*p* = 0.5
BSI (−1–1)	0.20 (0.27)	0.16 (0.23)	*p* = 0.6
English EIT (Max 120)	112.39 (6.83)	113.98 (4.82)	*p* = 0.11
Spanish EIT (Max 120)	105.56 (13.14)	53.65 (24.29)	***p* < 0.001**
Dominance (−210–210)	39.40 (45.26)	128.38 (33.85)	***p* < 0.001**
^*1*^ Mean (SD), ^*2*^ Two Sample t-test			

#### Correlational analysis.

Correlational analyses were performed to examine relationships between working memory, cognitive control, and syntactic processing for each of the bilingual groups. The results are presented in separate tables for each group: Table 2 shows the correlations for the L2 learner group, while Table 3 presents the correlations for the heritage speaker group. We will first discuss the results for Spanish syntactic processing, followed by English syntactic processing.

**Table 2:** Pearson correlation coefficients of individual difference and proficiency measures (Learners).

	BSI	OSPAN	EIT.English	EIT.Spanish	Dominance
BSI	1.00	−0.05	0.14	−0.06	0.05
OSPAN	−0.05	1.00	0.22	0.47***	−0.17
EIT.English	0.14	0.22	1.00	−0.09	0.22
EIT.Spanish	−0.06	0.47***	−0.09	1.00	−0.56***
		[0.20, 0.66]			
Dominance	0.05	−0.17	0.22	−0.56***	1.00
				[-0.72, -0.32]	

Note. **Bold** signifies p > 0.001. Confidence intervals included only on significant correlations.

**Table 3:** Pearson correlation coefficients of individual difference and proficiency measures (Heritage speakers).

	BSI	OSPAN	EIT.English	EIT.Spanish	Dominance
BSI	1.00	0.13	−0.04	−0.15	−0.13
OSPAN	0.13	1.00	0.52***	0.05	0.26
EIT.English	−0.04	0.52***	1.00	−0.24	**0.45**
		[0.23, 0.72]			[0.14,0.67]
EIT.Spanish	−0.15	0.05	−0.24	1.00	−0.48***
					[-0.69, -0.17]
Dominance	−0.13	0.26	0.45***	−0.48***	1.00

Note. **Bold** signifies p > 0.001. Confidence intervals included only on significant correlations.

**Fig 2:**
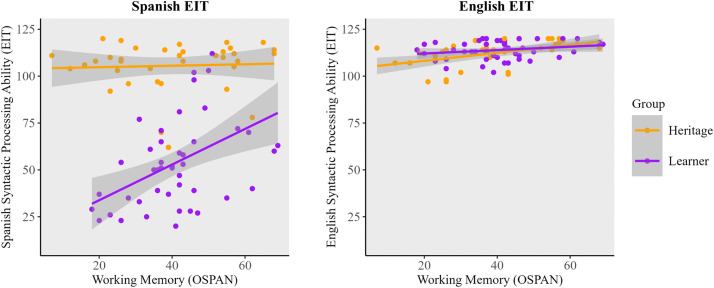
The relationship between working memory and syntactic processing.

**Spanish Syntactic Processing.** For L2 learners, analyses revealed medium-sized statistically significant positive correlations between Spanish syntactic processing and working memory. These relationships can be seen illustrated in Fig 2. No significant correlation was found between Spanish syntactic processing and cognitive control (BSI score). For heritage speakers, there was a medium-sized, statistically significant positive correlation between Spanish syntactic processing and dominance. However, no significant correlation was found between working memory and Spanish syntactic processing, nor between cognitive control and Spanish syntactic processing.

**Fig 3:**
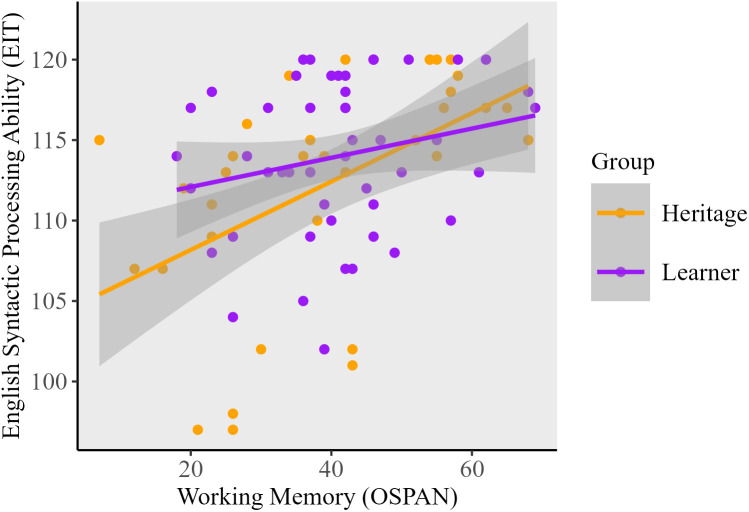
The relationship between working memory and English syntactic processing ability.

**English Syntactic Processing.** L2 Learners did not show any significant correlations between English syntactic processing and working memory, cognitive control (BSI score), or dominance. For heritage speakers, analyses revealed a large-sized, statistically significant positive correlation between working memory and English syntactic processing. This is illustrated in Fig 2. Regarding Fig 2, it is important to note that the visual representation of this correlation may appear less pronounced due to the consistent y-axis scaling used across both panels to facilitate direct cross-language comparison. A clearer illustration of the relationship between working memory and English syntactic processing is shown in Fig 3. As with Spanish, cognitive control was not significantly correlated with English syntactic processing.

The overall results show that learners’ Spanish syntactic processing was correlated with working memory capacity. Heritage speakers did not show effects of working memory on Spanish syntactic processing, but a strong correlation was found between working memory and English syntactic processing. The absence of cognitive control effects overall was unexpected. We return to discuss this later in the analysis and consider how the overall effects on the EIT may have masked cognitive control differences for sentences that were more syntactically complex. Next, we report results from linear mixed effects models on syntactic processing for both groups. All of these models can be found in S1 Appendix. Given that Spanish syntactic processing is far more variable in both groups, and thus more susceptible to the influence of both cognitive resources, it will be examined first.

### Spanish Syntactic Processing – L2 Learners

The maximal model estimates for Spanish syntactic processing for L2 learners are shown in Table 4 in S1 Appendix. First, the model yielded a significant main effect of working memory (β = 0.31, *SE* = 0.09 *t* = 3.60*, p* < .001), which indicates that L2 learners with higher working memory exhibited greater Spanish syntactic processing ability. Next, there was a significant main effect of dominance (β = −0.43, *SE* = 0.09 *t* = −4.53*, p* < .001). This indicates that L2 learners who reported being more English dominant exhibited decreased syntactic processing ability in the L2, Spanish. Contrary to the predictions, there were no significant effects of cognitive control. We had predicted that learners, for whom the use of Spanish and English are largely separate, would show a pattern of reactive cognitive control. The aggregated performance on the Spanish EIT did not support that prediction.

To further explore the more fine-grained components of language experience captured by the BLP, eight additional models were built to examine the influence of language history, language use, self-reported proficiency, and language attitudes in both English and Spanish. These models can be viewed in (Table 8–Table 15) in S1 Appendix. Overall, the main effect of working memory found in the maximal model remained constant across these analyses. No other significant interactions were found between the subcomponents of the BLP and the cognitive factors in the current study.

### Spanish Syntactic Processing – Heritage Speakers

The maximal model estimates for Spanish syntactic processing for heritage speakers are shown in Table 5 in S1 Appendix. The model yielded a significant main effect of dominance (β = −0.24, *SE* = 0.06 *t* = *−*3.97*, p* < .001), which indicates that heritage speakers who reported being more English dominant exhibited decreased syntactic processing ability in the heritage language, Spanish. Contrary to predictions, there were no significant effects of working memory or cognitive control on Spanish syntactic processing for heritage speakers.

To further explore the more fine-grained components of language experience for the heritage speakers, eight additional models were built to examine the influence of language history, language use, self-reported proficiency, and language attitudes in both English and Spanish. These models can be viewed in (Table 24–Table 31) in S1 Appendix. Here, a significant negative interaction was found between English language history and working memory (β = −0.16, *SE* = 0.06 *t* = −2.60*, p* < .001) (Table 24 in S1 Appendix). This suggests that the effect of working memory on Spanish syntactic processing ability is conditional on a heritage speaker’s history of English use. Specifically, for heritage speakers with a greater history of English use during childhood, the positive relationship between working memory and Spanish syntactic processing performance is weaker or less apparent. No other significant interactions were found between the subcomponents of the BLP and the cognitive factors in the current study.

### English Syntactic Processing – L2 Learners

The maximal model estimates for English syntactic processing for L2 learners are shown in Table 6 in S1 Appendix. The model yielded a significant main effect of dominance (β = 0.05, *SE* = 0.02 *t* = 2.43*, p* < .001), which indicates that L2 learners who reported being more English dominant exhibited greater syntactic processing ability in English. No other significant effects were found. More fine-grained analyses on the influence of language history, language use, self-reported proficiency, and language attitude (Table 16– Table 23 in S1 Appendix) revealed no significant interactions between these subcomponents of the BLP and the cognitive factors in the current study.

### English Syntactic Processing – Heritage Speakers

**Fig 4:**
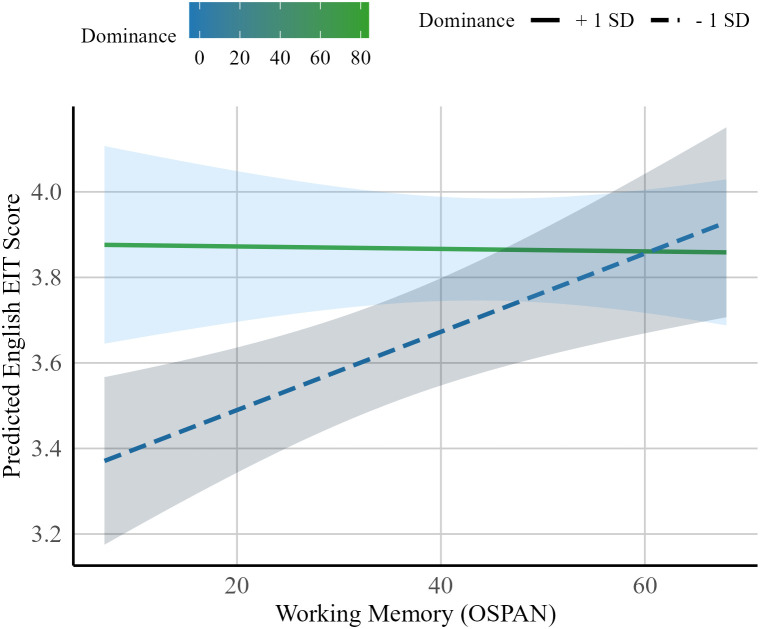
Interaction between dominance and working memory on English EIT scores (Heritage Speakers).

The maximal model estimates for English syntactic processing for heritage speakers are shown in Table 7 in S1 Appendix. First, the model revealed a main effect of working memory (β = 0.07, *SE* = 0.03 *t* = 2.43*, p* < .001), which suggests that heritage speakers with higher working memory exhibited increased syntactic processing ability in the societal language, English. Next, a significant main effect of dominance was found (β = 0.10, *SE* = 0.03 *t* = 3.18*, p* < .001). This suggests that heritage speakers who reported being more English dominant exhibited increased syntactic processing ability in English. Finally, a significant interaction was also found between working memory and language dominance (β = −0.08, *SE* = 0.03 *t* = −2.29*, p* < .001), which suggests that the positive effect of working memory on English syntactic processing for heritage speakers diminished as their English dominance increased. Put reciprocally, working memory seemed to play a more important role for heritage speakers who were more Spanish dominant and less English dominant. This interpretation can be seen visually below in Fig 4, where heritage speakers who are more Spanish dominant (blue line) show a more positive effect of working memory when compared to heritage speakers who are more English dominant (green line).

To further explore the interaction between working memory and dominance, eight additional models were built to examine the influence of language history, language use, self-reported proficiency, and language attitudes in both English and Spanish (Table 32– Table 39 in S1 Appendix). First, the model examining history of English use (Table 32 in S1 Appendix) revealed a significant interaction between working memory and history of English use (β = −0.07, *SE* = 0.03 *t* = −2.17*, p* = .037). This suggests that for heritage speakers with a greater history of English use during childhood, the positive relationship between working memory and English syntactic processing performance is weaker or less apparent. An additional interaction was also found between working memory and Spanish attitude (Table 39 in S1 Appendix) (β = 0.09, *SE* = 0.03 *t* = 3.03*, p* = .005). This suggests that the positive effect of working memory on English syntactic processing ability was increased for heritage speakers who reported a closer cultural attachment and emotional connection to Spanish. No other significant interactions were found between the subcomponents of the BLP and the cognitive factors in the current study.

### Complex Phrase Analyses: A measure of processing difficulty

The results of the current study have demonstrated an effect of working memory for learners processing Spanish. For heritage speakers, working memory effects were found when processing the societal language (English) and this effect was modulated by language dominance. However, contrary to our predictions, there were no effects of cognitive control for either group.

To examine the role of cognitive control more closely, post-hoc analyses were carried out to examine the hypothesis that these effects may only appear when the language processing demands are high. For the EIT, this analysis focused on the effect of phrase complexity. Although they need to be taken with caution, these additional analyses were motivated by a number of studies which report more robust cognitive effects only when the linguistic demands of a task are particularly high [64]. Sentences with more complex structure may require additional linguistic analysis and the requirement to briefly store information in memory. The question we ask in this post-hoc analysis is whether cognitive control and working memory become more critical as phrases become more complex.

Phrase complexity in the EIT was captured by an increase in syllable length. To help justify the use of syllable length as the phrase complexity metric, four linear mixed-effects regression models were built which specifically examined the effect of syllable length on syntactic processing in English and Spanish across both groups. Each model yielded a main effect of syllable length, indicating that as phrase syllable length increased, performance on the task decreased significantly. Subsequent analyses were carried out similar to the overall results, which provided the opportunity to directly compare effects of working memory and cognitive control on overall syntactic processing when considering increasing phrase complexity.

#### Complex Phrase Analysis: Spanish EIT.

First, the impact of phrase complexity in the Spanish EIT was examined. Separate models were created for both groups. The learner model can be seen in Table 40 in S1 Appendix. This model yielded significant main effects of working memory and syllable length, as well as a significant interaction between syllable length and working memory (β = 0.04, SE = 0.01 t = 4.55, p < .001). This indicates that working memory modulated the relationship between syllable length and Spanish syntactic processing such that the negative effect of syllable length on Spanish syntactic processing was attenuated for learners with higher levels of working memory. The interaction between syllable length and cognitive control was not significant. Next, the heritage speaker model can be seen in Table 41 in S1 Appendix. This model yielded significant main effects of syllable length and dominance, as well as an interaction between syllable length and dominance. Critically, this model revealed a significant interaction between syllable length and cognitive control (β = −0.01, *SE* = 0.006 *t* = *−*2.83*, p* = .004). As a reminder, cogni*t*ive control was computed using the Behavioral Shift Index (BSI), which ranges on a scale of −1–1, with negative values representing more reactive control and positive values representing more proactive control. The interaction in this model indicated that heritage speakers who exhibited more reactive control strategies were better able to process Spanish as phrase complexity increased.

#### Complex Phrase Analysis: English EIT.

We then analyzed the effect of phrase complexity in the English EIT. The model for learners can be seen in Table 42 in S1 Appendix. This model yielded a main effect of syllable length as well as a significant interaction between syllable length and cognitive control (β = 0.009, *SE* = 0.004 *t* = 2.3, *p* = .02). This interaction indicates that learners who engaged in more proactive control strategies (higher BSI score) were better able to process the more complex and demanding English EIT phrases. Next, the model for heritage speakers can be seen in Table 43 in S1 Appendix. This model yielded main effects of syllable length, dominance, and working memory. Overall, these results pattern similarly to the model examining overall English syntactic processing, which revealed positive effects of working memory and dominance for heritage speakers. Finally, no effects of cognitive control were found in the complex English EIT analysis for heritage speakers.

## Discussion

The current study examined two very different groups of speakers of English and Spanish to better understand how cognitive resources are recruited to support syntactic processing. Results revealed complex cross-group and cross-linguistic variability in how working memory and cognitive control were utilized to support language processing. First, working memory capacity was found to predict Spanish syntactic processing for L2 learners, but not for heritage speakers. Although both groups exhibited similar overall levels of English syntactic processing, only heritage speakers revealed a significant effect of working memory capacity on English syntactic processing – this effect was found to be modulated by language dominance, and, more specifically, by history of English use and attitude towards Spanish. Finally, a complex phrase analysis revealed unique effects of cognitive control for each group. First, heritage speakers who exhibited more reactive control strategies were better able to process Spanish as phrase complexity increased. This effect was not found for learners. Learners who exhibited more proactive control strategies were better able to process English as phrase complexity increased, and this effect was not found for heritage speakers. These findings will be discussed in more detail below, starting with working memory.

### Role of working memory

There exists ample evidence that supports the importance of working memory capacity during bilingual language processing [19–23]. Results from the current study align with this past work in showing that working memory predicted syntactic processing in both learners and heritage speakers. Notably, results additionally revealed considerable cross-linguistic and group level variation in how working memory was utilized to support language processing – learners revealed working memory effects during Spanish processing while heritage speakers revealed working memory effects during English processing. This complexity suggests that bilinguals with varied linguistic experiences may utilize their cognitive resources in different ways to support language use. But language experience is a broad term; and a question that arises is: what specific aspects of language experience might be driving this variation? In the current study, language experience was operationalized using a measure of language dominance, which captures variability in language history, proficiency, use, and attitudes in English and Spanish. By unpacking dominance into its core components, we can better explain why working memory effects emerge in distinct ways for learners and heritage speakers: proficiency appears to be most critical for learners, while history of English language use and attitudes towards Spanish appears more predictive for heritage speakers.

To help clarify the role of proficiency in modulating working memory engagement, consider the study’s results for Spanish syntactic processing. First, results for L2 learners revealed working memory effects during Spanish syntactic processing, but not for English processing. This pattern likely reflects their relatively lower proficiency in Spanish, which places higher demands on cognitive resources to maintain and integrate syntactic representations. Heritage speakers, in contrast, did not exhibit effects of working memory during the syntactic processing of Spanish. One way to interpret these asymmetrical effects is to consider differences in proficiency; heritage speakers exhibited much higher proficiency in Spanish as compared to L2 learners (both self-reported and objectively measured on the EIT) and thus may not have needed to draw upon working memory resources to perform what is likely a more automatized form of language processing. But for learners, lower Spanish proficiency likely increased the cognitive demand of syntactic processing, leading to stronger reliance on working memory. While this interpretation aligns with past research which suggests that working memory is engaged to support resource demanding processes, like the storage and maintenance of less automatized syntactic representations [21,22,58], it cannot account for group differences in working memory engagement for two groups of similar proficiency. This is a pattern of results we found in the current study when examining English syntactic processing. Below, we present an interpretation which suggests that variation in components of language experience, not only proficiency, can help explain the observed differences in working memory engagement during English syntactic processing.

Despite similar levels of English proficiency (Fig 1), only heritage speakers revealed effects of working memory during English syntactic processing, and these effects were modulated by language dominance. Heritage speakers who were more Spanish-dominant relied more on working memory resources when processing syntactic information in English. But what does “more” Spanish dominance represent for a heritage speaker? While for some it can reflect reduced proficiency in English, it can also reflect reduced patterns of past and current English language use. In the current study, it was found that one subcomponent of language dominance—history of English use—had a significant impact on working memory engagement during English syntactic processing. Specifically, working memory effects were decreased for heritage speakers who reported a greater history of English use during childhood. While this result should be interpreted with caution, it does suggest that the larger interaction found between working memory and Spanish-dominant heritage bilinguals (Table 7 in S1 Appendix) may be a reflection of their reduced use of English during childhood, and, more specifically, the less robust English representations that are a product of that reduced use.

Given that working memory involves the retrieval and active maintenance of task-relevant information alongside the suppression of competing representations [24,25], it follows that Spanish-dominant heritage speakers (who used English less during childhood) may engage working memory more intensely during English syntactic processing to both 1) support the retrieval of less robust English linguistic information and 2) manage interference from Spanish. We note as well that even the Spanish-dominant heritage speakers in the current study were living in an English-dominant environment, meaning that these Spanish-dominant bilinguals were continually required to use English, increasing the need to allocate cognitive resources. Although consequences of heritage experience for the home language have been the focus of much of the past research, the present study suggests that cognitive resources may be similarly important for supporting the maintenance and use of the societal language.

While our current design does not allow for a direct test of the varied cognitive mechanisms underlying working memory, and any interpretation remains speculative, one potential account, drawing on existing theoretical work, is that working memory mechanisms may be differentially recruited across groups. For example, the working memory measure used in the current study, the Operation Span (OSPAN) task, is a complex span task that captures both the maintenance of information under concurrent processing demands (storage) and the exertion of controlled attention to manage interference [24,25]. For L2 learners, working memory effects in Spanish may largely reflect the storage component, as processing less automatized syntactic structures increases demands on maintaining and integrating representations in real time [58]. Here, the OSPAN could be indexing learners’ ability to maintain syntactic representations.

By contrast, for heritage speakers, working memory effects in English could possibly reflect controlled-attention mechanisms engaged to regulate interference from highly activated Spanish representations during English processing. Because the OSPAN also taps attentional control, it is particularly well-suited to capture this regulatory engagement, suggesting that heritage speakers’ working memory effects reflect active suppression of Spanish during the retrieval and maintenance of less robust English representations. Critically, these effects emerged only in the most Spanish-dominant heritage speakers, consistent with the idea that greater activation of Spanish representations increases demands on controlled attentional resources during English syntactic processing. Ultimately, confirming this interpretation would require a multi-task approach to distinguish between storage and attentional control mechanisms more directly.

Another interpretation is that both mechanisms captured by the OSPAN task can explain the working memory effects in heritage speakers. That is, heritage speakers with higher working memory (particularly those who were Spanish dominant) demonstrated improved English syntactic processing because they were better able to both maintain the goal of English language use as well as implement some form of attentional control to combat interference. This same interpretation could be adopted for working memory effects found during Spanish syntactic processing for L2 learners; that is, learners with higher working memory were better able to both maintain the goal of processing Spanish representations, as well as implement attentional control to combat interference from the dominant language (English). This aligns with the conceptualization of working memory as a hierarchy of attentional control mechanisms [80]. Importantly, however, because the current study only employed a single measure of working memory and did not utilize a battery of measures to capture more fine-grained mechanisms of attention, inhibition, updating, and switching, these interpretations should be taken with caution. Ultimately, the working memory effects in the current study provide further evidence implicating language experience in shaping cognitive engagement during language use, contrary to the binary and homogenous classifications of bilinguals, a practice which has unfortunately been typical in research on language and cognition over the past several decades.

### Role of Cognitive Control

While effects of cognitive control on dual language use have been examined more frequently in proficient bilinguals [1,4], the current study adds to the emerging literature reporting control effects for language learners, individuals with far less experience using two languages [50]. The cognitive control effects found for L2 learners were related to syntactic processing in English, the L1, not Spanish, the L2. This outcome might seem counterintuitive because English was the more proficient and dominant language for the L2 learners. Crucially, the effect of proactive control was only observed in interaction with sentence complexity: higher proactive control facilitated processing, specifically for the more complex sentences in English. The control pattern we observed in this syntactic task is precisely what has been reported in the literature at other levels of language processing. Bilinguals learn to down-regulate their more dominant language to enable language processing in the less dominant language. Although it might seem that the more established and proficient language (English) would be less demanding of cognitive resources than the less proficient target language (Spanish), the evidence from a wide array of studies suggests otherwise. Evidence for the regulation of the dominant language comes from studies of lexical processing [4], lexical prediction in sentence context [26], and from performance on bilingual code-switching tasks [81]. See Kroll and Dussias [7] for a recent review. The recent work suggests that the L1 in learners begins to change in response to the presence of the L2 almost immediately [82]. The present findings extend the evidence for dynamic shifts in the dominant language under a broad range of language environments. The proactive control effects found in English for learners is enabling them to maintain their English proficiency while acquiring Spanish as an L2.

The results for heritage speakers add evidence to recent literature highlighting the dynamic ways in which heritage speakers recruit cognitive control to support dual language use [18]. In their study, Hernandez Santacruz et al. found that heritage speakers who relied on proactive control strategies produced lower accuracy and slower reaction times during a Spanish language production task, but higher accuracy and faster reaction times during an English language production task. This effect also varied as a function of the order in which participants switched between languages; proactive control strategies resulted in a decrease in accuracy when switching from Spanish to English, whereas no specific control strategy resulted in more accuracy when switching from English to Spanish. Although the tasks and modalities differ between Hernandez Santacruz et al. and the current study (language switching during production vs. syntactic processing), both studies converge on the observation that heritage speakers flexibly recruit cognitive control depending on contextual demands. In Hernandez Santacruz et al., extraneous task demands (the order of switching) shaped whether proactive control facilitated performance. In the current study, we found that reactive control strategies were recruited to support Spanish syntactic processing, but this effect was only found for the most complex sentences. Taken together, these findings suggest that heritage speakers’ reliance on cognitive control is not fixed but engaged dynamically to adapt to the linguistic and contextual pressures of the task at hand.

Crucially, the present study also provides new evidence for cognitive control effects at the sentence level, extending beyond prior studies focused on lexical production. We used the EIT because it allows both learners and more proficient bilingual speakers to perform in both languages. Examining sentence production outside of an imitation paradigm could have prevented learners, and potentially some heritage speakers, from generating adequate data. This is important because the EIT ensured comparability across groups, allowing for a controlled examination of cognitive engagement. Because both L2 learners and heritage speakers of varied proficiency can successfully engage with the EIT, the resulting data reflect meaningful differences in syntactic processing, rather than disparities in task feasibility. This allowed us to ask meaningful questions about how these different bilingual groups engage cognition to support language use. Had we used some form of spontaneous production task, it is likely that many participants in the current study would have struggled to complete it, making it very difficult to draw any strong conclusions regarding cross-group differences in cognitive engagement. Finally, examining the more syntactically complex phrases in the EIT revealed cognitive control effects even though the task did not require mapping new ideas to speech, suggesting that the effects we report likely underestimate the role of cognitive resources in producing well-formed sentences in each language.

We have argued that the pattern of reactive control reported for heritage speakers in the current study is related to the separate contexts in which they utilize their two languages. This aligns with theories of adaptive control [2] which hypothesize that bilinguals adapt to the linguistic demands of their environment. However, the reactive control pattern for heritage speakers arose only when examining the most syntactically complex phrases of the EIT. In the EIT, participants cannot anticipate the syntactic complexity of each sentence. Unlike language-switching paradigms with explicit cues that allow proactive preparation, the EIT presents sentences of varying complexity without warning, requiring rapid deployment of cognitive control to resolve interference and support language processing. We suggest that these task demands can help explain the reactive control effects; specifically, heritage speakers who deployed reactive control strategies, defined as a ‘late correction’ mechanism that is mobilized only as needed, in a just-in-time manner, may have been better able to manage the unexpected demands that arose during the processing of the more complex and unexpected phrases of the EIT task. This interpretation is supported by an alternative hypothesis, and one that is not necessarily mutually exclusive with the claim that interactional context chapes cognitive engagement, which is that these control effects are related to the difficulty of the task itself. Rivera et al. [83] recently reported that L2 learners with less proactive control profiles were better able to learn more difficult grammar rules. Rivera et al. suggests that more proactive mechanisms may potentially be advantageous in situations where the information is easy to keep and maintain active; however, when the information is more difficult or unexpected, less proactive control may better facilitate learning.

Although the Rivera et al. study focused on learning rather than online processing, the underlying mechanism may be similar: when information is complex or unpredictable, reactive control may better support adaptation to task demands. In their study, Rivera et al. argue that this idea could be related to working memory overload, which is in line with past research relating low working memory capacity with worse goal-maintenance performance [41,43]. However, the current results complicate a working memory-based interpretation: heritage speakers did not show evidence of working memory overload, even during the processing of the most syntactically complex phrases in Spanish. Despite this, however, they still employed a reactive control strategy. We suggest that this may reflect not only the difficulty of the linguistic input, but also the unpredictability of when such difficulty will occur. From this perspective, the use of reactive control is not necessarily a reflection of limited capacity, but rather an adaptive response to a task context in which proactive control is neither efficient nor feasible. This interpretation aligns with the Dual Mechanisms of Control framework [35], which emphasizes that cue reliability, rather than task difficulty alone, plays a critical role in determining whether individuals engage in proactive or reactive control. Thus, the reactive control observed in heritage speakers may reflect a strategic and efficient deployment of control under conditions of unpredictability, rather than a breakdown in goal maintenance due to cognitive overload.

Overall, the results from the current study largely support the predictions outlined in the introduction. For working memory, we anticipated that L2 learners would rely more heavily on this cognitive resource during Spanish syntactic processing and that heritage speakers would show working memory effects in English, modulated by language dominance. This pattern was confirmed, illustrating that working memory engagement during syntactic processing depends not only on proficiency or task difficulty, but also on each group’s unique language experiences. Regarding cognitive control, we predicted that learners would rely on reactive control mechanisms during Spanish processing, whereas heritage speakers might exhibit less of a clear reliance on one control strategy over the other. Here, the findings partially support these predictions. Heritage speakers exhibited reactive control strategies during the processing of complex Spanish phrases, aligning with the idea of separated language use and increased task demands, while learners showed proactive control strategies during the processing of complex English phrases.

## Conclusions

The current study explored the effects of language experience on the engagement of working memory and cognitive control during syntactic processing in English and Spanish across two bilingual groups: L2 learners of Spanish and English-Spanish heritage speakers. By examining two very different groups of language users, we showed that effects of working memory and cognitive control contribute to language processing in distinct ways. Together, these results contribute to our understanding of the effects of language experience on cognitive resources and shed new light on the dynamics that underlie variation in syntactic processing among bilinguals. These findings are of theoretical interest but also relevant to both second language and heritage language educators. Differences in cognitive engagement may reflect different avenues of learning that, if explicitly considered, might potentially result in enhanced linguistic achievement. We view the relationship between language experience and cognition to be a vital component for future studies seeking to better understand language processing. Our results go beyond accounts that focus on age of acquisition or proficiency alone to show that the context and history of language use is critical in shaping language processing and the cognitive resources that support it.

## Supporting information

S1 Appendix Analysis tables 4–47. (PDF)
