# Supplementary material for: Not just two languages: Using variation in language experience to understand how cognitive resources shape syntactic processing
Source: PLoS One. 2026 Apr 10;21(4):e0346505. doi: 10.1371/journal.pone.0346505 (PMC13068260; doi:10.1371/journal.pone.0346505)
Supplement: S1 Appendix — (PDF) [file pone.0346505.s001.pdf]

Supporting information

S1 Appendix. Analysis tables 4-47.

Full Maximal Models

Table 1 - Maximal Model - Estimated coefficients for Spanish syntactic processing (L2 Learners)

Effect	Estimate	*SE*	*t*	*p*
(Intercept)	1.76	0.17	10.61	**< .001**
Working Memory	0.31	0.09	3.60	**< .001**
Dominance	-0.43	0.09	-4.53	**< .001**
Cognitive Control	-0.01	0.09	-0.11	.916
Working Memory x Dominance	-0.13	0.13	-1.03	.307
Cognitive Control x Dominance	0.03	0.09	0.30	.763
Random Effects	Variance	SD		
Intercept | Participant	0.32	0.56		
Intercept | Item	0.59	0.77		

Note. Total *N* = 48. SE = standard error of the estimate. OSPAN, BSI, and Dominance were standardized (*z*-scores).

Table 2 - Maximal Model- Estimated coefficients for Spanish syntactic processing (heritage speakers)

Effect	Estimate	*SE*	*t*	*p*
(Intercept)	3.51	0.08	44.87	**< .001**
Working Memory	0.10	0.06	1.62	.113
Dominance	-0.24	0.06	-3.97	**< .001**
Cognitive Control	-0.10	0.06	-1.72	.094
Working Memory x Dominance	0.02	0.07	0.34	.733
Cognitive Control x Dominance	-0.05	0.07	-0.66	.516
Random Effects	Variance	SD		
Intercept | Participant	0.11	0.33		
Intercept | Item	0.37	0.61		

Note. Total *N* = 36. SE = standard error of the estimate. OSPAN, BSI, and Dominance were standardized (*z*-scores).

Table 3 - Maximal Model- Estimated coefficient for English syntactic processing (L2 learners)

Effect	Estimate	*SE*	*t*	*p*
(Intercept)	3.83	0.04	99.11	**< .001**
Working Memory	0.02	0.02	0.94	.353
Dominance	0.05	0.02	2.43	**.020**
Cognitive Control	0.03	0.02	1.31	.198

Working Memory x Dominance	0.01	0.03	0.19	.848
Cognitive Control x Dominance	-0.02	0.02	-1.07	.291
Random Effects	Variance	SD		
Intercept | Participant	0.12	0.33		
Intercept | Item	0.37	0.61		

Note. Total $N = 48$. SE = standard error of the estimate. OSPAN, BSI, and Dominance were standardized (*z*-scores).

Table 4 - Maximal Model - Estimated coefficients for English syntactic processing (heritage speakers)

Effect	Estimate	*SE*	*t*	*p*
(Intercept)	3.77	0.06	68.46	**< .001**
Working Memory	0.07	0.03	2.43	**.021**
Dominance	0.10	0.03	3.18	**.003**
Cognitive Control	-0.01	0.03	-0.21	.836
Working Memory x Dominance	-0.08	0.03	-2.29	**.029**
Cognitive Control x Dominance	-0.02	0.03	-0.47	.640
Random Effects	Variance	SD		
Intercept | Participant	0.02	0.14		
Intercept | Item	0.05	0.23		

Note. Total $N = 36$. SE = standard error of the estimate. OSPAN, BSI, and Dominance were standardized (*z*-scores).

BLP Subcomponent Analysis

L2 learners – Spanish syntactic processing

Table 5 - Estimated coefficients for the role of English language history on Spanish syntactic processing (L2 learners)

Effect	Estimate	*SE*	*t*	*p*
(Intercept)	1.80	0.17	10.32	**< .001**
Working Memory	0.33	0.11	3.10	**.003**
English History	-0.12	0.10	-1.18	.244
Cognitive Control	-0.05	0.11	-0.51	.610
Working Memory x English History	0.08	0.10	0.80	.425
Cognitive Control x English History	0.04	0.10	0.37	.710
Random Effects	Variance	SD		
Intercept | Participant	0.009	0.02		
Intercept | Item	0.001	0.03		

Note. Total $N = 48$. SE = standard error of the estimate. OSPAN, BSI, and English History were standardized (*z*-scores).

Table 6 - Estimated coefficients for the role of Spanish language history on Spanish syntactic processing (L2 learners)

Effect	Estimate	*SE*	*t*	*p*
(Intercept)	0.90	0.07	13.12	**< .001**
Working Memory	0.34	0.05	3.23	**.002**
Spanish History	0.06	0.05	0.68	.501
Cognitive Control	-0.04	0.04	-0.40	.692
Working Memory x Spanish History	0.02	0.04	0.50	.621
Cognitive Control x Spanish History	0.04	0.05	0.90	.374
Random Effects	Variance	SD		
Intercept | Participant	0.46	0.68		
Intercept | Item	0.60	0.77		

Note. Total $N = 48$. SE = standard error of the estimate. OSPAN, BSI, and Spanish History were standardized (*z*-scores).

Table 7 - Estimated coefficients for the role of English language use on Spanish syntactic processing (L2 learners)

Effect	Estimate	*SE*	*t*	*p*
(Intercept)	0.92	0.06	14.68	**< .001**
Working Memory	0.42	0.04	4.14	**< .001**
English Use	-0.25	0.06	-2.30	.061
Cognitive Control	-0.08	0.04	-1.20	.408
Working Memory x English Use	-0.16	0.06	-1.45	.154
Cognitive Control x English Use	-0.06	0.04	-0.59	.560
Random Effects	Variance	SD		
Intercept | Participant	0.40	0.63		
Intercept | Item	0.60	0.77		

Note. Total $N = 48$. SE = standard error of the estimate. OSPAN, BSI, and English Use were standardized (*z*-scores).

Table 8 - Estimated coefficients for the role of Spanish language use on Spanish syntactic processing (L2 learners)

Effect	Estimate	*SE*	*t*	*p*
(Intercept)	1.78	0.17	10.26	**< .001**

Working Memory	0.42	0.10	4.11	**< .001**
Spanish Use	0.18	0.12	1.45	.152
Cognitive Control	-0.01	0.10	-0.15	.884
Working Memory x Spanish Use	0.17	0.13	1.32	.195
Cognitive Control x Spanish Use	-0.09	0.12	-0.77	.445
Random Effects	Variance	SD		
Intercept | Participant	0.44	0.66		
Intercept | Item	0.60	0.77		

Note. Total N = 48. SE = standard error of the estimate. OSPAN, BSI, and Spanish Use were standardized (z-scores).

Table 9 - Estimated coefficients for the role of English self-rated proficiency on Spanish syntactic processing (L2 learners)

Effect	Estimate	*SE*	*t*	*p*
(Intercept)	1.78	0.17	10.22	**< .001**
Working Memory	0.30	0.11	2.79	**.008**
English Proficiency	0.08	0.10	0.81	.420
Cognitive Control	-0.05	0.11	-0.42	.674
Working Memory x English Proficiency	-0.04	0.11	-0.33	.746
Cognitive Control x English Proficiency	-0.16	0.10	-1.55	.128
Random Effects	Variance	SD		
Intercept | Participant	0.45	0.67		
Intercept | Item	0.60	0.77		

Note. Total N = 48. SE = standard error of the estimate. OSPAN, BSI, and English Self-Rated Proficiency were standardized (z-scores).

Table 10 - Estimated coefficients for the role of Spanish self-rated proficiency on Spanish syntactic processing (L2 learners)

Effect	Estimate	*SE*	*t*	*p*
(Intercept)	1.78	0.17	10.39	**< .001**
Working Memory	0.30	0.10	3.05	**.004**
Spanish Proficiency	0.30	0.10	3.12	**.003**
Cognitive Control	-0.01	0.09	-0.14	.892
Working Memory x Spanish Proficiency	0.04	0.11	0.34	.739
Cognitive Control x Spanish Proficiency	-0.07	0.09	-0.81	.421
Random Effects	Variance	SD		
Intercept | Participant	0.45	0.67		

Intercept | Item	0.60	0.77

Note. Total *N* = 48. SE = standard error of the estimate. OSPAN, BSI, and Spanish Self-Rated Proficiency were standardized (*z*-scores).

Table 11 - Estimated coefficients for the role of English attitudes on Spanish syntactic processing (L2 learners)

Effect	Estimate	*SE*	*t*	*p*
(Intercept)	1.79	0.17	10.31	**< .001**
Working Memory	0.30	0.11	2.97	**.005**
English Attitude	0.01	0.10	0.06	.953
Cognitive Control	-0.09	0.10	-0.84	.403
Working Memory x English Attitude	-0.06	0.10	-0.61	.542
Cognitive Control x English Attitude	-0.02	0.10	-2.33	.78
Random Effects	Variance	SD		
Intercept | Participant	0.44	0.67		
Intercept | Item	0.60	0.77		

Note. Total *N* = 48. SE = standard error of the estimate. OSPAN, BSI, and English Attitude were standardized (*z*-scores).

Table 12 - Estimated coefficients for the role of Spanish attitudes on Spanish syntactic processing (L2 learners)

Effect	Estimate	*SE*	*t*	*p*
(Intercept)	1.74	0.18	9.91	**< .001**
Working Memory	0.20	0.10	2.81	**.007**
Spanish Attitude	0.29	0.10	2.90	**.006**
Cognitive Control	0.02	0.10	0.23	.818
Working Memory x Spanish Attitude	0.07	0.13	0.53	.601
Cognitive Control x Spanish Attitude	-0.13	0.09	-1.40	.167
Random Effects	Variance	SD		
Intercept | Participant	0.44	0.67		
Intercept | Item	0.60	0.77		

Note. Total *N* = 48. SE = standard error of the estimate. OSPAN, BSI, and Spanish Attitude were standardized (*z*-scores).

BLP Subcomponent Analysis

L2 learners – English syntactic processing

Table 13 - Estimated coefficients for the role of English language history on English syntactic processing (L2 learners)

Effect	Estimate	*SE*	*t*	*p*
(Intercept)	3.86	0.04	96.39	**< .001**
Working Memory	0.02	0.02	1.11	.274
English History	0.08	0.03	3.03	**.003**
Cognitive Control	0.04	0.02	1.94	.058
Working Memory x English History	-0.01	0.02	-0.62	.541
Cognitive Control x English History	-0.02	0.02	-0.79	.434
Random Effects	Variance	SD		
Intercept | Participant	0.01	0.11		
Intercept | Item	0.02	0.16		

Note. Total $N = 48$. SE = standard error of the estimate. OSPAN, BSI, and English History were standardized (z-scores).

Table 14 - Estimated coefficients for the role of Spanish language history on English syntactic processing (L2 learners)

Effect	Estimate	*SE*	*t*	*p*
(Intercept)	3.81	0.04	95.16	**< .001**
Working Memory	0.01	0.02	0.34	.736
Spanish History	-0.04	0.03	-1.28	.207
Cognitive Control	0.04	0.02	1.79	.082
Working Memory x Spanish History	-0.03	0.02	-1.68	.100
Cognitive Control x Spanish History	0.00	0.02	-0.11	.910
Random Effects	Variance	SD		
Intercept | Participant	0.01	0.11		
Intercept | Item	0.02	0.17		

Note. Total $N = 48$. SE = standard error of the estimate. OSPAN, BSI, and Spanish History were standardized (z-scores).

Table 15 - Estimated coefficients for the role of English language use on English syntactic processing (L2 learners)

Effect	Estimate	*SE*	*t*	*p*
(Intercept)	3.84	0.04	95.85	**< .001**
Working Memory	0.01	0.02	0.67	.509
English Use	-0.04	0.03	-1.48	.145

Cognitive Control	0.03	0.02	1.28	.207
Working Memory x English Use	-0.01	0.03	-0.24	.810
Cognitive Control x English Use	0.02	0.02	0.96	.343
Random Effects	Variance	SD		
Intercept | Participant	0.01	0.11		
Intercept | Item	0.02	0.17		

Note. Total *N* = 48. SE = standard error of the estimate. OSPAN, BSI, and English Use were standardized (*z*-scores).

Table 16 - Estimated coefficients for the role of Spanish language use on English syntactic processing (L2 learners)

Effect	Estimate	*SE*	*t*	*p*
(Intercept)	3.83	0.04	98.50	**< .001**
Working Memory	0.00	0.02	0.07	.946
Spanish Use	-0.04	0.02	-1.73	.091
Cognitive Control	0.03	0.02	1.34	.187
Working Memory x Spanish Use	-0.03	0.02	-1.29	.206
Cognitive Control x Spanish Use	0.01	0.02	0.43	.671
Random Effects	Variance	SD		
Intercept | Participant	0.01	0.11		
Intercept | Item	0.02	0.17		

Note. Total *N* = 48. SE = standard error of the estimate. OSPAN, BSI, and Spanish Use were standardized (*z*-scores).

Table 17 - Estimated coefficients for the role of English self-rated proficiency on English syntactic processing (L2 learners)

Effect	Estimate	*SE*	*t*	*p*
(Intercept)	3.88	0.04	96.61	**< .001**
Working Memory	0.03	0.02	1.46	.152
English Proficiency	-0.10	0.03	-3.77	**< .001**
Cognitive Control	0.03	0.02	1.77	.083
Working Memory x English Proficiency	0.02	0.02	0.84	.403
Cognitive Control x English Proficiency	0.02	0.02	1.23	.223
Random Effects	Variance	SD		
Intercept | Participant	0.01	0.11		
Intercept | Item	0.02	0.17		

Note. Total *N* = 48. SE = standard error of the estimate. OSPAN, BSI, and English Self-Rated Proficiency were standardized (*z*-scores).

Table 18 - Estimated coefficients for the role of Spanish self-rated proficiency on English syntactic processing (L2 learners)

Effect	Estimate	*SE*	*t*	*p*
(Intercept)	3.85	0.04	98.92	**< .001**
Working Memory	0.03	0.02	1.36	.180
Spanish Proficiency	-0.07	0.02	-3.28	**.002**
Cognitive Control	0.03	0.02	1.73	.091
Working Memory x Spanish Proficiency	0.02	0.02	1.10	.277
Cognitive Control x Spanish Proficiency	0.01	0.02	0.57	.572
Random Effects	Variance	SD		
Intercept | Participant	0.01	0.11		
Intercept | Item	0.02	0.17		

Note. Total *N* = 48. SE = standard error of the estimate. OSPAN, BSI, and Spanish Self-Rated Proficiency were standardized (*z*-scores).

Table 19 - Estimated coefficients for the role of English attitudes on English syntactic processing (L2 learners)

Effect	Estimate	*SE*	*t*	*p*
(Intercept)	3.85	0.04	95.12	**< .001**
Working Memory	0.02	0.02	1.00	.322
English Attitude	-0.05	0.03	-2.07	**.042**
Cognitive Control	0.04	0.02	1.70	.097
Working Memory x English Attitude	0.02	0.02	0.85	.398
Cognitive Control x English Attitude	0.01	0.02	0.64	.527
Random Effects	Variance	SD		
Intercept | Participant	0.01	0.11		
Intercept | Item	0.02	0.17		

Note. Total *N* = 48. SE = standard error of the estimate. OSPAN, BSI, and English Attitude were standardized (*z*-scores).

Table 20 - Estimated coefficients for the role of Spanish attitudes on English syntactic processing (L2 learners)

Effect	Estimate	*SE*	*t*	*p*
(Intercept)	3.85	0.04	98.19	**< .001**
Working Memory	0.03	0.02	1.62	.112
Spanish Attitude	-0.08	0.02	-3.76	**< .001**

Cognitive Control	0.02	0.02	1.01	.317
Working Memory x Spanish Attitude	0.01	0.03	0.45	.652
Cognitive Control x Spanish Attitude	0.01	0.02	0.74	.464
Random Effects	Variance	SD		
Intercept | Participant	0.01	0.11		
Intercept | Item	0.02	0.17		

Note. Total $N = 48$. SE = standard error of the estimate. OSPAN, BSI, and Spanish Attitude were standardized (*z*-scores).

BLP Subcomponent Analysis

Heritage speakers Spanish syntactic processing

Table 21 - Estimated coefficients for the role of English language history on Spanish syntactic processing (Heritage)

Effect	Estimate	*SE*	*t*	*p*
(Intercept)	3.50	0.07	49.56	**< .001**
Working Memory	0.01	0.05	0.21	.832
English History	-0.33	0.06	-5.35	**< .001**
Cognitive Control	-0.05	0.05	-0.94	.352
Working Memory x English History	-0.16	0.06	-2.60	**.014**
Cognitive Control x English History	-0.04	0.06	-0.68	.504
Random Effects	Variance	SD		
Intercept | Participant	0.07	0.28		
Intercept | Item	0.07	0.26		

Note. Total $N = 36$. SE = standard error of the estimate. OSPAN, BSI, and English History were standardized (*z*-scores).

Table 22 - Estimated coefficients for the role of Spanish language history on Spanish syntactic processing (Heritage)

Effect	Estimate	*SE*	*t*	*p*
(Intercept)	3.52	0.09	40.26	**< .001**
Working Memory	0.04	0.07	0.56	.581
Spanish History	0.04	0.07	0.49	.628
Cognitive Control	-0.07	0.07	-0.95	.348
Working Memory x Spanish History	0.03	0.08	0.41	.682
Cognitive Control x Spanish History	0.01	0.08	0.09	.929

Random Effects	Variance	SD
Intercept | Participant	0.07	0.28
Intercept | Item	0.07	0.26

Note. Total *N* = 36. SE = standard error of the estimate. OSPAN, BSI, and Spanish History were standardized (*z*-scores).

Table 23 - Estimated coefficients for the role of English language use on Spanish syntactic processing (Heritage)

Effect	Estimate	*SE*	*t*	*p*
(Intercept)	3.51	0.08	42.62	**< .001**
Working Memory	0.04	0.07	0.66	.512
English Use	-0.17	0.07	-2.51	**.017**
Cognitive Control	-0.11	0.07	-1.57	.125
Working Memory x English Use	0.10	0.07	1.48	.148
Cognitive Control x English Use	-0.01	0.07	-0.22	.824
Random Effects	Variance	SD		
Intercept | Participant	0.07	0.28		
Intercept | Item	0.07	0.26		

Note. Total *N* = 36. SE = standard error of the estimate. OSPAN, BSI, and English Use were standardized (*z*-scores).

Table 24 - Estimated coefficients for the role of Spanish language use on Spanish syntactic processing (Heritage)

Effect	Estimate	*SE*	*t*	*p*
(Intercept)	3.50	0.08	41.96	**< .001**
Working Memory	0.06	0.07	0.93	.358
Spanish Use	0.16	0.07	2.34	**.025**
Cognitive Control	-0.10	0.07	-1.42	.164
Working Memory x Spanish Use	-0.07	0.07	-0.91	.370
Cognitive Control x Spanish Use	0.01	0.08	0.18	.858
Random Effects	Variance	SD		
Intercept | Participant	0.07	0.28		
Intercept | Item	0.07	0.26		

Note. Total *N* = 36. SE = standard error of the estimate. OSPAN, BSI, and Spanish Use were standardized (*z*-scores).

Table 25 - Estimated coefficients for the role of English self-rated proficiency on Spanish syntactic processing (Heritage)

Effect	Estimate	*SE*	*t*	*p*

(Intercept)	3.52	0.08	41.47	**< .001**
Working Memory	0.03	0.07	0.47	.641
English Proficiency	0.09	0.07	1.29	.205
Cognitive Control	-0.06	0.07	-0.92	.366
Working Memory x English Proficiency	0.03	0.07	0.45	.657
Cognitive Control x English Proficiency	0.05	0.07	0.62	.539
Random Effects	Variance	SD		
Intercept | Participant	0.15	0.39		
Intercept | Item	0.07	0.26		

Note. Total $N = 36$. SE = standard error of the estimate. OSPAN, BSI, and English Self-Rated Proficiency were standardized (*z*-scores).

Table 26 - Estimated coefficients for the role of Spanish self-rated proficiency on Spanish syntactic processing (Heritage)

Effect	Estimate	*SE*	*t*	*p*
(Intercept)	3.52	0.08	44.47	**< .001**
Working Memory	0.05	0.06	0.85	.402
Spanish Proficiency	0.20	0.06	3.11	**.004**
Cognitive Control	-0.07	0.06	-1.02	.316
Working Memory x Spanish Proficiency	0.05	0.06	0.70	.487
Cognitive Control x Spanish Proficiency	0.07	0.07	1.01	.319
Random Effects	Variance	SD		
Intercept | Participant	0.12	0.35		
Intercept | Item	0.07	0.26		

Note. Total $N = 36$. SE = standard error of the estimate. OSPAN, BSI, and Spanish Self-Rated Proficiency were standardized (*z*-scores).

Table 27 - Estimated coefficients for the role of English attitudes on Spanish syntactic processing (Heritage)

Effect	Estimate	*SE*	*t*	*p*
(Intercept)	3.52	0.09	40.40	**< .001**
Working Memory	0.03	0.07	0.44	.663
English Attitude	0.04	0.07	0.58	.566
Cognitive Control	-0.07	0.07	-0.90	.372
Working Memory x English Attitude	0.02	0.07	0.30	.765
Cognitive Control x English Attitude	0.04	0.07	0.50	.617

Random Effects	Variance	SD
Intercept | Participant	0.16	0.40
Intercept | Item	0.07	0.26

Note. Total *N* = 36. SE = standard error of the estimate. OSPAN, BSI, and English Attitude were standardized (*z*-scores).

Table 28 - Estimated coefficients for the role of Spanish attitudes on Spanish syntactic processing (Heritage)

Effect	Estimate	*SE*	*t*	*p*
(Intercept)	3.51	0.08	42.03	**< .001**
Working Memory	0.05	0.07	0.74	.467
Spanish Attitude	0.15	0.07	2.16	**.038**
Cognitive Control	-0.08	0.07	-1.10	.277
Working Memory x Spanish Attitude	0.00	0.07	0.04	.967
Cognitive Control x Spanish Attitude	0.06	0.07	0.87	.388
Random Effects	Variance	SD		
Intercept | Participant	0.44	0.67		
Intercept | Item	0.60	0.77		

Note. Total *N* = 36. SE = standard error of the estimate. OSPAN, BSI, and Spanish Attitude were standardized (*z*-scores).

BLP Subcomponent Analysis

Heritage speakers English syntactic processing

Table 29 - Estimated coefficients for the role of English language history on English syntactic processing (Heritage)

Effect	Estimate	*SE*	*t*	*p*
(Intercept)	3.77	0.05	70.26	**< .001**
Working Memory	0.11	0.03	3.81	**< .001**
English History	0.10	0.04	2.56	**.014**
Cognitive Control	-0.01	0.03	-0.35	.732
Working Memory x English History	-0.07	0.03	-2.17	**.037**
Cognitive Control x English History	0.01	0.04	0.35	.730
Random Effects	Variance	SD		
Intercept | Participant	0.02	0.14		
Intercept | Item	0.05	0.23		

Note. Total N = 36. SE = standard error of the estimate. OSPAN, BSI, and English History were standardized (z-scores).

Table 30 - Estimated coefficients for the role of Spanish language history on English syntactic processing (Heritage)

Effect	Estimate	*SE*	*t*	*p*
(Intercept)	3.76	0.06	65.63	**< .001**
Working Memory	0.11	0.03	3.23	**.003**
Spanish History	0.07	0.04	1.81	.078
Cognitive Control	-0.03	0.03	-1.06	.298
Working Memory x Spanish History	-0.06	0.04	-1.64	.110
Cognitive Control x Spanish History	-0.01	0.04	-0.39	.700
Random Effects	Variance	SD		
Intercept | Participant	0.03	0.17		
Intercept | Item	0.05	0.23		

Note. Total N = 36. SE = standard error of the estimate. OSPAN, BSI, and Spanish History were standardized (z-scores).

Table 31 - Estimated coefficients for the role of English language use on English syntactic processing (Heritage)

Effect	Estimate	*SE*	*t*	*p*
(Intercept)	3.74	0.06	67.85	**< .001**
Working Memory	0.09	0.03	3.15	**.004**
English Use	0.10	0.03	3.33	**.002**
Cognitive Control	0.00	0.03	-0.05	.957
Working Memory x English Use	-0.06	0.03	-2.03	.051
Cognitive Control x English Use	0.00	0.03	-0.13	.901
Random Effects	Variance	SD		
Intercept | Participant	0.03	0.14		
Intercept | Item	0.05	0.23		

Note. Total N = 36. SE = standard error of the estimate. OSPAN, BSI, and English Use were standardized (z-scores).

Table 32 - Estimated coefficients for the role of Spanish language use on English syntactic processing (Heritage)

Effect	Estimate	*SE*	*t*	*p*
(Intercept)	3.76	0.06	67.24	**< .001**

Working Memory	0.08	0.03	2.54	**.016**
Spanish Use	-0.09	0.03	-2.60	**.014**
Cognitive Control	-0.01	0.03	-0.22	.824
Working Memory x Spanish Use	0.05	0.03	1.65	.110
Cognitive Control x Spanish Use	0.00	0.03	0.06	.950
Random Effects	Variance	SD		
Intercept | Participant	0.03	0.14		
Intercept | Item	0.05	0.23		

Note. Total *N* = 36. SE = standard error of the estimate. OSPAN, BSI, and Spanish Use were standardized (*z*-scores).

Table 33 - Estimated coefficients for the role of English self-rated proficiency on English syntactic processing (Heritage)

Effect	Estimate	*SE*	*t*	*p*
(Intercept)	3.77	0.06	64.75	**< .001**
Working Memory	0.11	0.03	3.14	**.003**
English Proficiency	-0.08	0.04	-1.77	.081
Cognitive Control	-0.02	0.03	-0.65	.522
Working Memory x English Proficiency	0.06	0.03	1.78	.084
Cognitive Control x English Proficiency	-0.01	0.03	-0.35	.730
Random Effects	Variance	SD		
Intercept | Participant	0.03	0.14		
Intercept | Item	0.05	0.23		

Note. Total *N* = 36. SE = standard error of the estimate. OSPAN, BSI, and English Self-Rated Proficiency were standardized (*z*-scores).

Table 34 - Estimated coefficients for the role of Spanish self-rated proficiency on English syntactic processing (Heritage)

Effect	Estimate	*SE*	*t*	*p*
(Intercept)	3.78	0.06	66.84	**< .001**
Working Memory	0.09	0.03	2.81	**.008**
Spanish Proficiency	-0.08	0.04	-1.92	.060
Cognitive Control	-0.01	0.03	-0.41	.685
Working Memory x Spanish Proficiency	0.06	0.03	1.96	.058
Cognitive Control x Spanish Proficiency	-0.04	0.03	-1.20	.239
Random Effects	Variance	SD		
Intercept | Participant	0.03	0.14		

Intercept | Item	0.05	0.23		

Note. Total *N* = 36. SE = standard error of the estimate. OSPAN, BSI, and Spanish Self-Rated Proficiency were standardized (*z*-scores).

Table 35 - Estimated coefficients for the role of English attitudes on English syntactic processing (Heritage)

Effect	Estimate	*SE*	*t*	*p*
(Intercept)	3.75	0.06	63.60	**< .001**
Working Memory	0.09	0.04	2.68	**.012**
English Attitude	0.00	0.04	-0.02	.984
Cognitive Control	-0.02	0.03	-0.55	.586
Working Memory x English Attitude	0.02	0.04	0.68	.502
Cognitive Control x English Attitude	-0.03	0.04	-0.92	.364
Random Effects	Variance	SD		
Intercept | Participant	0.03	0.14		
Intercept | Item	0.05	0.23		

Note. Total *N* = 36. SE = standard error of the estimate. OSPAN, BSI, and English Attitude were standardized (*z*-scores).

Table 36 - Estimated coefficients for the role of Spanish attitudes on English syntactic processing (Heritage)

Effect	Estimate	*SE*	*t*	*p*
(Intercept)	3.80	0.06	68.12	**< .001**
Working Memory	0.10	0.03	3.34	**.002**
Spanish Attitude	-0.10	0.04	-2.73	**.008**
Cognitive Control	-0.03	0.03	-1.01	.319
Working Memory x Spanish Attitude	0.09	0.03	3.03	**.005**
Cognitive Control x Spanish Attitude	-0.04	0.03	-1.55	.131
Random Effects	Variance	SD		
Intercept | Participant	0.02	0.14		
Intercept | Item	0.05	0.23		

Note. Total *N* = 36. SE = standard error of the estimate. OSPAN, BSI, and Spanish Attitude were standardized (*z*-scores).

Complex models - Spanish

Table 37 - Estimated coefficients for Spanish syntactic processing (Learners, complex model)

Effect	Estimate	*SE*	*t*	*p*
(Intercept)	0.88	0.04	17.46	**< .001**
Syllable Length	-0.24	0.03	-7.48	**< .001**
Working Memory	0.15	0.03	3.88	**< .001**
Cognitive Control	-0.009	0.03	-0.24	0.810
Syllable Length x Working Memory	0.04	0.01	4.55	**< .001**
Syllable Length x Cognitive Control	-0.004	0.00	-1.09	0.27
Random Effects	Variance	SD		
Intercept | Participant	0.07	0.24		
Intercept | Item	0.02	0.16		

Note. Total $N = 48$. SE = standard error of the estimate. Dependent variable is log-transformed Spanish EIT score. Syllable Length, OSPAN, and BSI, were standardized (*z*-scores).

Table 38 - Estimated coefficients for Spanish syntactic processing (Heritage speakers, complex model)

Effect	Estimate	*SE*	*t*	*p*
(Intercept)	1.48	0.01	76.23	**< .001**
Syllable Length	-0.04	0.01	-4.63	**< .001**
Dominance	-0.07	0.01	-3.69	**< .001**
Working Memory	0.02	0.01	1.35	0.18
Cognitive Control	-0.03	0.01	-1.84	0.07
Syllable Length x Dominance	-0.02	0.006	-4.51	**< .001**
Syllable Length x Working Memory	0.005	0.006	0.90	0.36
Syllable Length x Cognitive Control	-0.01	0.006	-2.83	**.004**
Random Effects	Variance	SD		
Intercept | Participant	0.06	0.09		
Intercept | Item	0.02	0.04		

Note. Total $N = 36$. SE = standard error of the estimate. Dependent variable is log-transformed Spanish EIT score. Syllable Length, Dominance, OSPAN, and BSI, were standardized (*z*-scores).

Complex models – English

Table 39 - Estimated coefficients for English syntactic processing (Learners, complex model)

Effect	Estimate	*SE*	*t*	*p*
(Intercept)	1.58	0.01	160.95	**< .001**
Syllable Length	-0.02	0.008	-2.99	**< .001**
Working Memory	0.005	0.005	1.04	0.30
Cognitive Control	0.009	0.005	1.6	0.10
Syllable Length x Working Memory	0.004	0.004	1.06	0.29
Syllable Length x Cognitive Control	0.009	0.004	2.31	**0.02**
Random Effects	Variance	SD		
Intercept | Participant	0.001	0.02		
Intercept | Item	0.001	0.03		

Note. Total $N = 48$. SE = standard error of the estimate. Dependent variable is log-transformed Spanish EIT score. Syllable Length, OSPAN, and BSI, were standardized (*z*-scores).

Table 40 - Estimated coefficients for English syntactic processing (Heritage speakers, complex model)

Effect	Estimate	*SE*	*t*	*p*
(Intercept)	1.57	0.01	113	**< .001**
Syllable Length	-0.04	0.01	-3.51	**0.002**
Dominance	0.02	0.008	2.93	**0.006**
Working Memory	0.01	0.008	2.1	**0.04**
Cognitive Control	0.000	0.007	0.04	0.9
Syllable Length x Dominance	0.006	0.006	1.06	0.2
Syllable Length x Working Memory	0.011	0.006	1.88	0.06
Syllable Length x Cognitive Control	-0.004	0.005	-0.8	0.4
Random Effects	Variance	SD		
Intercept | Participant	0.001	0.03		
Intercept | Item	0.002	0.04		

Note. Total $N = 36$. SE = standard error of the estimate. Dependent variable is log-transformed Spanish EIT score. Syllable Length, Dominance, OSPAN, and BSI, were standardized (*z*-scores).

Appendix B

Table 41 - EIT Scoring Criteria

Score	Criteria
4	Perfect repetition
3	Meaning preserved; use of synonyms or (grammatical or ungrammatical) changes in grammar that do not affect meaning
2	More than half of the content preserved; slight changes in content that make content inexact, incomplete, or ambiguous
1	Half or less of content repeated; important content is left out; meaning may be unrelated or opposed to stimulus
0	Silence, unintelligible content, or only one content word.

Table 42 - Distribution of Syllable Length Spanish EIT Stimuli

Number of Syllables	Count of EIT Sentences with this Syllable Length
7	2
9	3
10	1
11	1
12	3
13	2
14	2
16	6
16	4
17	2
18	4

Table 43 - Distribution of Syllable Length English EIT Stimuli

Number of Syllables	Count of EIT Sentences with this Syllable Length
7	1
8	2
9	1
10	2
11	1
12	3
13	2
14	3
15	4
16	5
17	2
18	3
19	1

Table 44 - Correlations between English EIT item number and several measures of syntactic complexity

	Sentence Number (Item)
Words	**0.878**
Sentences	NA
Verb Phrases	**0.418**
Clauses	**0.488**
T-Units	NA
Dependent Clauses	**0.538**
Complex T-units	**0.495**
Complex Phrases	0.079
Complex Nominals	0.269
Mean Length of Sentence	**0.878**
Mean Length of T-unit	**0.878**
Mean Length of Clause	0.109
Clauses per Sentence	**0.488**
Verb Phrases per T-unit	**0.418**
Clauses per T Unit	**0.488**
Dependent Clauses per Clause	**0.526**

Dependent Clauses per T-unit	**0.538**
T-units per Sentence	NA
Complex t-unit Ratio	**0.495**
Coordinate Phrases per T-unit	0.079
Coordinate Phrases per Clause	0.026
Complex Nominals per T-unit	0.269
Complex Nominals per Clause	0.053
Note. ***Bold*** signifies $p > 0.001$	
